# Control of basal autophagy rate by *vacuolar peduncle*

**DOI:** 10.1371/journal.pone.0209759

**Published:** 2019-02-08

**Authors:** Marc Bourouis, Magali Mondin, Aurore Dussert, Pierre Leopold

**Affiliations:** 1 Université Côte d’Azur, Centre National de la Recherche Scientifique, Institut de Biologie Valrose, Nice–France; 2 Université Côte d’Azur, Institut national de la santé et de la recherche médicale, Institut de Biologie Valrose, Nice -France; Univerzitet u Beogradu, SERBIA

## Abstract

Basal autophagy is as a compressive catabolic mechanism engaged in the breakdown of damaged macromolecules and organelles leading to the recycling of elementary nutrients. Thought essential to cellular refreshing, little is known about the origin of a constitutional rate of basal autophagy. Here, we found that loss of *Drosophila vacuolar peduncle* (*vap*), a presumed GAP enzyme, is associated with enhanced basal autophagy rate and physiological alterations resulting in a wasteful cell energy balance, a hallmark of overactive autophagy. By contrast, starvation-induced autophagy was disrupted in *vap* mutant conditions, leading to a block of maturation into autolysosomes. This phenotype stem for exacerbated biogenesis of PI(3)P-dependent endomembranes, including autophagosome membranes and ectopic fusions of vesicles. These findings shed new light on the neurodegenerative phenotype found associated to mutant *vap* adult brains in a former study. A partner of Vap, Sprint (Spri), acting as an endocytic GEF for Rab5, had the converse effect of leading to a reduction in PI(3)P-dependent endomembrane formation in mutants. *Spri* was conditional to normal basal autophagy and instrumental to the starvation-sensitivity phenotype specific of *vap*. Rab5 activity itself was essential for PI(3)P and for pre-autophagosome structures formation. We propose that Vap/Spri complexes promote a cell surface-derived flow of endocytic Rab5-containing vesicles, the traffic of which is crucial for the implementation of a basal autophagy rate.

## Introduction

Autophagy (formally macroautophagy) is a conserved self-eating process with homeostatic and cytoprotective functions important in many aspects of human health [1, 2]. Autophagy is often considered as an instrument for survival when eukaryotic cells or organisms are facing stress conditions. Virtually, the so-called ‘nonselective autophagy’ involves the sequestration of bulk cytoplasmic material, including organelles, into dedicated double-membrane compartments called autophagosomes, and the delivery of cargoes to the lysosomes for degradation. Therefore, autophagy assumes a major catabolic role for recycling.

In parallel to its induction by stress, a basal rate of autophagy is taking place constitutively in most cells for a renewal of damaged organelles or elimination of misfolded proteins or aggregates [3–5]. This cellular refreshing has an active role in preventing numbers of neurodegenerative diseases [6–8]. Basal autophagy also largely contributes to metabolic homeostasis and to the balancing of whole organism energy, promoting the heath of cells and tissues [9–11]. For examples, autophagy insures the constant degradation of lipid droplets in hepatocytes, a process called lipophagy [12, 13]. Estimates of global needs in energy in fact indicated that ca. 20% of the daily dietary-calories intake is burned just to renew structures degraded by autophagy turnover [9, 10]. Consistent, autophagy has a cost in energy, which is manifested by growth suppressive effects [14, 15]. It was proposed literally that autophagy and growth define antagonistic cellular fates [16].

Autophagy also has developmental importance. Aside from its critical role in the supply of autophagy nutrients in mammalian pre-implantation embryos and neonates ([2, 17] and refs therein), autophagy is required for the cytoplasmic remodeling of differentiating erythrocytes, lymphocytes and adipocytes [17]. Additionally, remodeling of entire tissues as in amphibians and insects, or tissue regression as in vertebrates, involve autophagy-based degeneration, which is fulfilled by a physiological cell death process called type II cell death [18]. While autophagy degeneration (or type II cell death) display characteristic cellular morphologies like the presence of abundant autolysosomes in dying cells, novel studies from both vertebrate and insect models showed that type II death can be Caspase-dependent, a feature mostly specific of the type I cell death program [19]. Indeed, autophagy death and the Caspase machinery were acting synergistically or sequentially so to achieve elimination of the complete cell mass ([19, 20] and refs therein). Autophagy-specific structures are massively amplified by starvation signaling. Studies of autophagosome biogenesis in such induced conditions led to the description of succeeding steps referred to as initiation and nucleation, expansion and completion, and finally maturation that is followed by the recycling of constituents, including lysosome reformation. Activities of a range of *ATG genes* (*AuTophagy Genes*) are coordinating advancement in the process [2, 3, 21]. Initiation is mediated by the nutritional sensing kinase Atg1/ULK1, which acts both as limiting factor and master trigger of autophagy induction and advancement, while being under repressive control by the biosynthetic mTOR kinase (mammalian Target of Rapamycin) [22–25].

Early autophagosome nucleating structures are visualized as phosphatidylinositol 3-phosphate-enriched (PI(3)P) sites found along ER protrusions known as omegasomes [26]. This network of nascent autophagosomes originates from activation of dormant Vps34 complexes (named for the constituting class III PI3-kinase), which relocate from a cell peripheral reservoir [27]. This is followed by on-site recruitment of downstream *ATG* proteins in the PAS (pre-autophagosome structures), which further expand in double-layered isolation membranes (IM) or phagophores by the action of two autophagy-specific ubiquitin-like systems [21]. Expansion and completion phases include the encapsulation of cargoes and end with vesicle closure [21]. Maturation of autophagosomes entails the supply of extraneous effectors proteins and hydrolytic enzymes in the degradation-active autophagolysosomes simply called autolysosomes. Not only this implies fusion events to the acidified lysosomes, but in higher eukaryotes this is preceded by series of fusions to vesicles of the endocytic pathway that are now recognized as essential for maturation [28, 29].

PI(3)P phosphoinositide happened as a crucial player for several classes of endomembrane identities and dynamics [30–32]. Classically, early endosomes generation relies on membrane-inserted Rab5-GTP that stimulates heterodimeric Vps34-Vps15/p150 effector recruitment, thus coupling PI(3)P formation to Rab5 localization [33]. Subsequent coordination between Rab5 and PI(3)P containing platforms attract a network of specialized effectors and regulators, ending into defined early endosomal membranes [32, 33]. A distinct complex, referred as the Vps34-core enzyme comprising Beclin1/Atg6 in addition to the catalytic Vps34-Vps15/p150 subunits, is cataloged as having proautophagic activity [34, 35]. Indeed, Vps34-complex I that includes Vps34-core proteins linked to the ER-targeting subunits Atg14, is being involved in early steps of autophagy membrane nucleation along omegasomes [36, 37]. Vps34-core also has later requirements for fusions of maturating autophagosomes [35, 36]. Interestingly, preceding work revealed a function for Rab5 in autophagy initiation and found the presence the GTPase in macromolecular complexes containing Beclin1 and Vps34 [38]. How Rab5 plays a role during autophagy membranes organization however remains unclear and controversial [39].

The *Drosophila melanogaster* larval fat body is a proven, genetically-tractable model and simple read-out for many autophagy issues as integrated in the living animal [3, 24, 40, 41]. Like many larval cells, nutrient-storing fat cells are committed to endoreplicative growth during feeding larval stages, which last for three 24h or 48h-long instars, while ending by sudden (hormonally-induced) autophagy degeneration program at 108h AEL (after egg lying). During this period and the subsequent days of metamorphic development, nutrients issued from the fat body stores act like key determinants of final adult body mass, health and fitness characteristics. While developmentally programmed autophagy is taking place in the late 3^rd^ post-feeding larvae, a distinct autophagy response referred to as ‘starvation-induced’, is executed by the larval fat bodies in event of nutrient shortage during growth periods. Time course of this stereotyped response is well established [42, 43], with new autophagy structures, including lysosomes, emerging in a 1h period [41, 43], whereas the number of autophagosomes is peaking and then replaced by the final degradation-active autolysosomes in a 4h period [42]. Whereas much of the induced-autophagy processes, including its regulation and developmental function, were being explored in the fly systems [3, 40], the bases of a constitutional basal autophagy and its physiological importance remains elusive.

In ongoing, fat body-directed, genetic screening, we uncovered RNAi(s) targeting the gene *vap*, a GTPase activating protein (GAP) ranked as a member of five *D*.*melanogaster* RasGAP relatives [44]. Incidentally, mutations at the *vap* locus were first identified as brain structural mutants (hence the name of *Vacuolar Peduncle*) and later associated with shortened lifespan owing to an age-related brain neurodegeneration phenotypes [45, 46]. In this work, we used the fat body autophagy system, to establish that mutant, *vap* exhibited deregulated basal autophagy rate. We found that alterations resulting from the absence of *vap*, originate in the endocytic compartment through the unchecked activity of Sprint, an activator of Rab5 and partner of Vap [47, 48]. Interestingly, RASA1 the human ortholog of Vap, was recovered previously in a survey of genes involved in endocytic trafficking [49]. This study for the first time brings genetic evidence linking endocytic Rab5-positive vesicles generation, their trafficking along the endolysosomal and autophagy pathways and basal autophagy rate establishment.

## Materials and methods

### Screening and isolation of *vap* RNAi

A search for modifiers of a fat body-dependent starvation phenotype was performed by crossing the tester line: *ppl-Gal4/ CyO*, *tub-Gal80; pUY-slif-a* (called *slif-anti)*, to a selection of one thousand RNAi-transgenic lines as chosen among genes found misregulated in the *slif-a* fat tissue. Targeted *slif-a* expression using a *ppl-Gal4* driver resulted in a robust nutrition-independent starvation phenotype when developed at 25°C [50]. This included developmental delay, reduction of larval size and death at the pupal stage. When developed at 18°C however, all animals emerged as small-sized adults [50]. Suppressor RNAi lines were selected for viability at 25°C, whereas enhancer RNAi lines were selected from the 18°C crosses as reducing adult viability and size, and were subsequently validated.

The *vap*-RNAi lines 9209 T1 III (id.44638) and 9209 KK (id.107341) (VDRC collection) enhanced the *slif-a*-induced phenotype. Such enhancement was recapitulated by heterozygosity at the locus, i.e. in *vap*^*1*^/+ flies. QRT-PCR analysis using RNA from dissected 3^rd^ instar larval fat bodies confirmed that *vap* transcripts were present in this tissue and that the RNAi lines 9209 T1 and 9209 KK lowered this expression by 3 fold of the *wt* level when driven with *cg-Gal4*.

### Fly stocks and genetics

The X-linked *vap*^*1*^, *vap*^*2*^ and *vap*^*3*^ alleles were all shown to dramatically affect Vap protein levels [46]. *vap*^*1*^ is a genetically null mutant, *vap*^*2*^ *a* near null and *vap*^*3*^ a strong hypomorph mutant [46]. All three mutants are mostly viable as adult and were kept as homozygous lines. Transgenic flies carrying *wt* or mutant, myc-tagged *Vap/RasGAP* constructs were described before [51]. These were: *UAS-Vap(wt) 16*.*4* (a wild-type protein), *UAS-Vap(R695K*) GAP* (a GAP catalytic domain point mutant), UAS-Vap(SH2*32*) 22.*2* (a two SH2-domains point mutants), UAS-Vap(SH2*32) N15.*1* (a first SH2-domain point mutant), UAS-Vap(SH23*2) B59.*1* (an SH3-domain point mutant) and *UAS-Vap(SH232) 17*.*3* (an none-mutated N-terminal fragment). A scheme of these constructs can be found in **S5 Fig.**

The X-linked *spri*^*6G1*^ mutant is small deletion and RNA-null mutant, thought viable [52]. GFP:Sprint fusion protein was expressed using an UASp-HA:GFP:Spri-a:6xHis plasmid [52], used to make transgenic lines (BestGene Inc.). These were identified as *UASp-GFP*:*Spri 7m*, *8m* and *9m*. Recombinant *vap*^*1*^, *FRT19A* chromosome was generated by standard method based on *neo*^***R***^ positive-selection of the *FRT* bearing chromosome. Recombinant *vap*^*2*^, *spri*^*6G1*^ was from [48]. The PI(3)P biosensor GFP:FYVE is carried by a *UAS-GFP*:*myc*:*2xFYVE* construct of ch3 or *UAS-myc*:*2xFYVE* of ch2 (a gift from M.Conzales-Gaëtan lab). The biosensor comprises two FYVE fingers domains of mouse Hrs, that binding exclusively to position-3 mono-phosphorylated phosphoinositides [53]. Recombinant strains *UAS-GFP*:*myc*:*2xFYVE*, *Act>CD2>GAL4* and *Act>CD2>GAL4*, *UAS-GFP*:*Atg8a* and *2xUAS-EGFP*, *FRT40A*, *Fb-GAL4; UAS-GFP*:*myc*:*2xFYVE* are described in [34], the p-*mChAtg8a* construct is described in [54]. Low level of ATG1 was expressed from the ch3 transgene *UAS-myc*:*Atg1* [55]. The *Unk-LacZ* reporter line is described in [56]. RNAi line, *Atg1(RI)* is from the DRSC/TRiP Harvard Medical School collection (P-TRiP.JF02273-ATG1), *Spri(RI)* from the VDRC KK collection (id.101164), and the *Atg5(RI)* line 5-24-1 is described in [43].

Other stocks were as in Flybase (http://flybase.org/): *Atg8a*^*1*^ line EP(1)262-Atg8a; *Atg8a*^*2*^ line KG07569-Atg8a; *UAS-GAP1* line RasGAP1-5, ch3; *UAS-NF1* line GAP-66A, ch3; *UAS-dVps34 (Pi3K59F)* line J-wt m7, ch2; *UAS-dVps34(DN)* line KD-m8, ch2; *UAS-Atg8a*:*GFP* line M4A, ch3; *Rab5*^*2*^ line k08230 DWL; *UAS-Rab5*:*GFP*, ch3; *UAS-Rab7*:*GFP*, ch2; *UAS-Rab5(DN)* line S43N, ch3; *UAS-Rab5(CA)* line Q88L, ch3.

The used driver lines are described in Flybase: *ppl-Gal4*.*P* (*pumpless-Gal4*); *cg-Gal4* (*cg25C/collagen type IV-Gal4*); *Act-Gal4*.*A* (*Actin5C-Gal4*); *arm-Gal4*.*S* (*armadillo-Gal4*); *Mhc-Gal4*.*W* (*MHC-82/myosin heavy chain-Gal4*); *elav-Gal4*.*L2* (*embryonic lethal abnormal vision-Gal4*).

#### Fly culture and growth standardization

When appropriate, larvae were staged by 0-6h egg-lay collections. Calibrated growth conditions were further realized by picking 20 individuals o the 24h-emerging larvae into single culture tubes. Standard food medium (containing 17g/l of dried yeast) was sometimes enriched by doubling the amount of dried yeast (2X medium or aa-rich food), or depleted to one third of dried yeast (0.3X medium or aa-poor food). The latter growth medium caused a chronic starvation state with notable developmental delay. Adult mass was determined using five days-old adult flies from calibrated cultures and pooling 15 flies per assay. Developmentally staged larval fat bodies were analyzed at respectively 72h (end-2L), 80-85h (early/mid 3L), 96h (mid 3L) or 110h (end 3L/wandering). Starvation-induced autophagy was achieved by transferring larvae to 0.8% agar in PBS buffer, 0% sucrose, referred as acute starvation conditions. Starvation period was optimized to focus on either autophagosome biogenesis stage [42], i.e. 1h30’-2h for early autophagy events and 3h-4h for steady-state autophagy process.

#### Starvation-sensitivity assays

Adult flies were collected from low density cultures or calibrated cultures, and aged further for 2–3 days at 18°C while fed, unless specified in the experiment. Assay for starvation sensitivity were performed in tube containing 0.8% agar in PBS buffer. In each experiment, viability was recorded using a minimum of 5 tubes of 20 flies each, and alive individuals counted 2 times a day or more often if flies were dying fast. Mean viability was plotted. Variability between tubes repetitions of same genotype rarely exceeded plus or minus 5 flies. For this reason and for clarity of the charts, error bars were omitted in the viability curves. Experiments were repeated 2–4 times to confirm the outcome of given experiments.

#### Generation of fat body clones

Flp-mediated, loss-of-function mitotic recombination clones of fat body cells were induced in collection of 0-8h embryos by heat shocking culture tubes in a 38°C water bath for 2h30’ both for the FRT40 and FRT19/MARCM bearing genotypes. Staged larvae of appropriate genotypes were picked and checked under the fluorescent dissecting microscope for potential recombination events, i.e. GFP-negative clones, 2XUAS-GFP sister clones or GFP-positive spots in larval tissues in case of MARCM labeling. The flipout cassette method was used for clonal expression of UAS- bearing transgenes to conduct g-o-f expression, DN-construct expression and RNAi silencing. Fat cells clones arose spontaneously in the prototypical genotypes: *hs-Flp; Act>CD2>Gal4*, *UAS-GFP*, *UAS-X*, where expression was obtained by *Act5C*>*Gal4* driver. Larvae with GFP-positive fat body clones were picked and prepared for mounting.

#### Live tissue analysis of GFP and LysoTracker fluorescence

When stated, fat body preparations were analyzed in live tissue to avoid fixation-prone artifacts, as follows: fat tissues with GFP-based markers were dissected in PBS and subjected to LysoTracker staining if needed. In this case, fat body pads were incubated for 2 min in 100 nM LysoTracker Red DND-99 /Green DND-26 (Molecular Probes) and 1 μM Hoechst 33342 in PBS [43]. Tissues were transferred in a drop of PBS or PBS in 80%glycerol on a microscope slide and covered with coverslips using spacers and sealed with nail polish for immediate imaging.

#### Immunofluorescent staining of fat bodies

Whole fat tissues were fixed in PBS, 4% formaldehyde (Sigma-Aldrich) for 30–45 min, rinsed and permeabilized in PBS, 0,3% triton for 5–10 min. Tissues were blocked in PBS 0,1% triton (PBT), 5% normal goat serum, 1% BSA for 2-14h at 20° or 4°C. Primary antibodies were incubated for overnight at 4°C, washed two to three times for 15 min in PBT and incubated with secondary antibodies for 2h at 20°C. If needed, washed tissues were stained with fluorescent phalloidin conjugate (FluoProbes) at 0.165 μM for 20 min, and nuclei counterstained in 1 μM Höescht (Sigma-Aldrich) for 1 min. Dissected fat body pads were mounted in 90% glycerol, 20 mM Tris-Hcl pH8.0, 0,5% n-propyl gallate and coverslips sealed with nail polish. Primary Antibodies: Anti-p62 /Ref(2)P (a gift from D. Contamine) 1:1000; anti-Avl (a gift from D.Bilder) 1:1000; anti-Hrs (a gift from H.Bellen’s lab) 1:500; anti-Rab5 (Abcam) 1:200–1:400; anti-C-Myc 9E10 (Santa Cruz Biotechnology) 1:1000; anti-βGalactosidase 1:1000 (GeneTex). Secondary antibodies were Cyanine conjugates or Alexa Fluor conjugates (Jackson Immunoresearch Laboratories or Molecular Probes/ Invitrogen) were used as recommended.

Fluid-phase endocytosis labeling of early endosomes was carried as in [34]. Dissected fat bodies were exposed for 25min to complete S2 medium containing 160 μg/ml Texas-Red avidin (Invitrogen) and then chased for 30min in the absence of tracer.

#### PI(3)P ELISA and TAG assays

Total lipid content of whole larvae was extracted by chloroform and enriched as an acidic lipids fraction starting from frozen collections of 10 larvae per sample, following an adaptation of the protocol used in the PI(3)P Mass ELISA Kit-K-3300 (Echelon). Competitive ELISA assays were run in triplicates to determine PI(3)P phosphoinositides content in the total acid lipid extracts. Absorbance at 450nm was measured on a TECAN-Sunrise plate reader. Concentrations were determined using calibrated standard of PI(3)P concentrations as established in the kit. Triacylglycerol (TAG) determination in adult flies was carried as in [57].

#### TEM analysis

Fat body pads of selected genotypes were dissected and fixed for greater that 14h at 4°C, in 1.5% glutaraldehyde, 0.075 M cacodylate buffer, insect isotonic. Tissues were post-fixed in 150 mM OsO4, 100 mM Na-phosphate buffer and dehydration steps were minimized to preserve lipid content before embedding in Epon according to standard procedures. 60–70 nanometer-thick sections were collected on 200 mesh copper grids, stained with uranyl acetate and viewed on a transmission electron microscope (120 kV JEOL JEM-1400). Pictures of vesicular organelles in fat bodies in both fed and starved animals of each control and *vap* mutant were compared. Semi-quantitative analysis of typical autophagy structures [58] of starved samples was conducted based on 66 (wt) and 68 (vap^1^) images obtained from fixed numbers of analyzed sections.

#### Microscopy

Confocal images were acquired on an Axiovert 200M inverted stand (Carl Zeiss Microscopy GmbH, Jena, Germany), using 25X NA0.8 multi immersion objectives, or 40X oil NA1.3. Alternatively a Zeiss LSM 710 microscope with inverted Axio Observer.Z1 stand was used, with LD-LCI Plan Apo 25X NA0.8 multi immersion objectives and Plan Apo 63X oil NA1.4, equipped with LASER diode 405 nm, Argon LASER (458, 488, 514 nm), DPSS 561 nm, and HeNe 633 nm. ZEN 2011 software (Carl Zeiss) was used for image acquisition.

#### Image analysis and statistics

Fiji or Photoshop softwares were used to assess signal superposition of distinct channels following segmentation, based on thresholds intensity. Channel specific binary masks were intersected and overlap determined as pixel numbers or area using “common pixels” or “histogram” functions. Overlapping areas for the Rab7:GFP/p62 or GFP:Atg8a/LysoTracker or Rab5/GFP:Spri signals were calculated after their respective thresholding, using ROI(s) for individual selected cells or particles and expressed relative to the p62 or LysoTracker or GFP:Spri stained areas respectively, using wide field images. Distribution of particles size and circularity shape descriptor for GFP:Atg8a /LysoTracker staining channels was performed on large field images (obj.25X zoom 4.0–5.0) that were segmented after intensity thresholding and then graphed. Evaluations of endocytic Rab5 and Avl vesicle densities were performed using large field images (obj.63X zoom 2.2) encompassing 2–3 cells diameters. Densities were expressed as the ratio of counted Rab5 or Avl vesicles after thresholding, to actual cellular areas (in μm^2^) taken form phalloidin stainings. Quantification of p62 bodies and Rab7:GFP aggregates was performed similarly but expressed as density per cell areas, using defined ROI in 7–13 individual cells chosen in selected images. Density and size of GFP:Atg8a-labeled vesicles in Rab5-CA expressing cells or wild-type control clonal cells were obtained similarly, summing vesicles of 2–3 Z-plans in 2–3 images each. Boxplots of LysoTracker, p62, Rab7:GFP staining particles/area were traced using the GraphPad Prism software, omitting whiskers. Excel (Microsoft) or Prism (GraphPad) software were used to evaluate normality of populations, and statistics applied using Student’s *t*-tests (parametric) or Mann Whitney test (non-parametric) to compared two populations. Parametric or non-parametric ANOVA compared more than two populations. Significant statistics used the following convention: * = p<0.05; ** = p<0.01; *** = p<0.001. Non-significant statistics: n.s. = (p> 0.05).

## Results

### I. Basal autophagy is modulated through *vap-*dependent PI(3P) regulation

Fat body-targeted inhibition of the Slimfast (*slif*) amino-acid (aa) transporter resulted in organism-wide starvation phenotypes with impact on body growth, nitrogen and protein turn-over, as well as lipid store remobilization [50, 59]. Based on this genetic paradigm, we search for modifier genes using biased RNAi screening (Materials and Methods) with the aim to find *slif*-subordinated processes. Silencing of *vap* identified it as a genuine enhancer of the *slif*-dependent starvation (Materials and Methods). Incidentally, previous interest about this gene concerned its fly-brain neurodegenerative phenotype, the origin of which was uncertain but was likely triggered by an autophagy cell death process [46].

**Fig 1:**
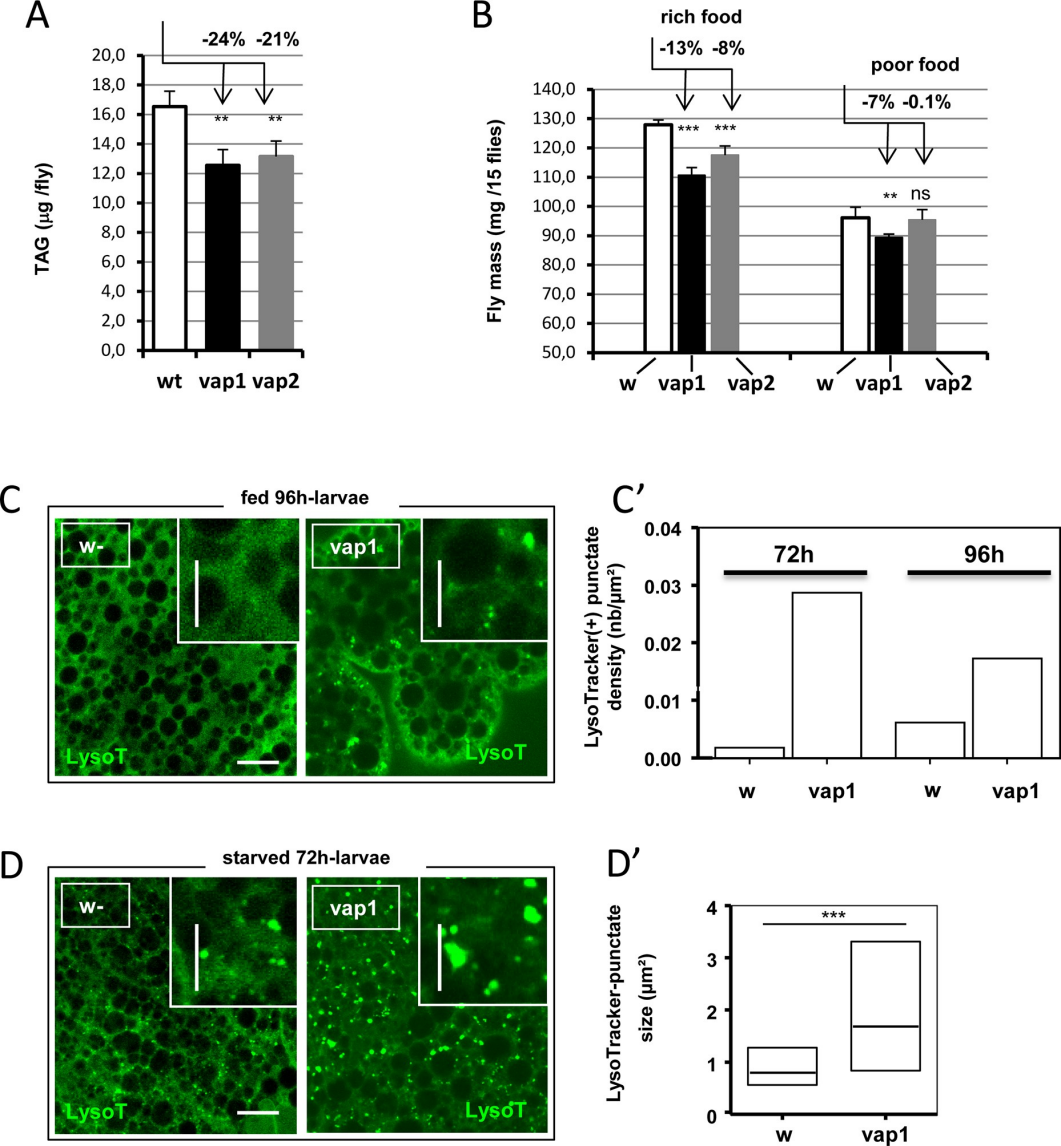
Perturbations of animal fitness and basal, or starvation-induced autophagy in *vap* mutants. (A) The total amount of TAG in aged-matched samples of adult male flies grown in rich food was compared between control, *w-* and mutant, *vap*^*1*^ and *vap*^*2*^ (n = 4). Mutants showed diminished TAG stores. (B) The mass of adults male flies was compared in aged-matched samples when grown in rich food or poor food (aa-rich or aa-poor conditions, Materials and Methods) (n = 5–8). Mutants had chronic mass deficits in rich conditions. Mutant flies emerged 10h in advance compared to controls but had quite limited mass deficits when grown in poor food conditions. The same effects were observed in females. In A-B, error bars are mean differences; significances are from Student’s *t*-tests. (C) Acidic lysosomal compartments were revealed by pH-sensitive LysoTracker staining in life tissue. Fat bodies in 96h, mid-3^rd^ *vap1* larvae but not control, *w-* showed punctuate fluorescent particles. Mutant fat bodies also exhibited reduced cell adhesion. Scale bar = 20 μm. Inset: higher magnification images. Scale bars = 10 μm. (C’) LysoTracker-positive punctate densities were evaluated on images obtained in C, using 72h and 96h samples (n = 2). Both time points showed abnormal occurrence of lysosomal staining in *vap*^*1*^. (D) Starvation-induced lysosomal response was analyzed in 72h, 2^nd^ instar larvae subjected to complete food deprivation for 3h. Fat bodies of control, *w-* showed frequent LysoTracker-positive vesicles but mutant, *vap*^*1*^ fat bodies elicited a stronger response, including the presence of many large acidic particles (compare image in insets; Scales as in C). Similar effects were seen in *vap*^*2*^ and *vap*^*3*^ mutants. (D’) The size distribution of LysoTracker positive punctate in experiment in D was quantified. The size of punctate particles of two wide field images obtained from identically stained tissues, counting 192 particles (*w-)* and 488 particles (*vap*^*1*^) were analyzed and graphed as boxplots. Mutant tissue shows particles spanning a larger size range (*w-*, Mdn = 0.80 μm^2^; *vap*^*1*^, Mdn = 1.68 μm^2^). See **S1A Fig** for complete size distribution of the particles. Medians are drawn as thick lines; significance is from Mann Whitney test. Genotypes. (A, B) Control: *w*^*1118*^*/Y*. Assay: *vap*^*1*^*/Y*. *vap*^*2*^*/Y*. (C, C’, D, D’) Control: *w*^*1118*^*/Y*. Assay: *vap*^*1*^*/Y*.

To establish a fat-specific role for *vap*, but also to elucidate the relationship between autophagy and neurodegeneration, we looked for simple alterations of fat body-dependent functions, including autophagy constants, taking advantage of currently available mutant of the gene that were all largely viable in adult flies (Materials and Methods). We first noticed lean fat bodies in larvae of mutant *vap*^*1*^, *a* null allele of the gene. This correlated with lower of triglycerides (TAG) reserves in the adult flies (**Fig 1A**). Significantly, we also observed a deficit of ca.10% of the adult body-mass in normally fed mutant animals (**Fig 1B**). Surprisingly, this mass deficit almost vanished if flies were grown in nutrient-limiting conditions when compared to controls treated similarly (**Fig 1B**). Furthermore, the loss of *vap* was not inducing any developmental delay, even under growth-limiting conditions (**Fig 1)**. Together, these features suggest that *vap* activity is mostly manifested in the fed animals and could thus be related to energy-metabolism regulation or fat-body store remobilization, rather than being contributing to TOR-dependent animal growth rate. Coherent with this, we found no obvious down regulation of TOR-signaling when this was assayed in fat bodies of mutant larvae (**S3C Fig**).

To inquire about the status of autophagy in the feeding larvae, we compared controls to *vap*^*1*^ mutant fat bodies in 96h, mid-3^rd^ instars, a stage before the onset of developmental autophagy at 108h AEL. As expected, we saw no lysosomes or autolysosomes structures in controls when revealed by LysoTracker dye staining. However, the 96h *vap*^*1*^ fat tissue showed distinctly stained lysosome-like structures (**Fig 1C**). The presence of lysosomes in fed mutant animals was confirmed in analyses of fat bodies in 72h, 2^nd^ instars and was quantified at the two time points (**Fig 1C’**). The early presence and persisting character of such acidic vesicles is in favor of a misregulation of the mutant autophagy system rather than an advancement of the programmed developmental autophagy. We thus looked at the starvation-induced lysosomal response using fat bodies of 72h larvae. In 3h-starved control animals, LysoTracker-positive lysosomes of a usual size (of ca. 0.8 μm) were induced. However, these acidic structures were greater in number and larger in size (of > 2.0 μm) in the mutant tissue (**Fig 1D and 1D’ and S1A Fig**). The intensified response in the starvation-induced context is compatible with the idea that the autophagy machinery is already primed in the fed fat cells, possibly because of abnormally elevated basal autophagy.

**Fig 2:**
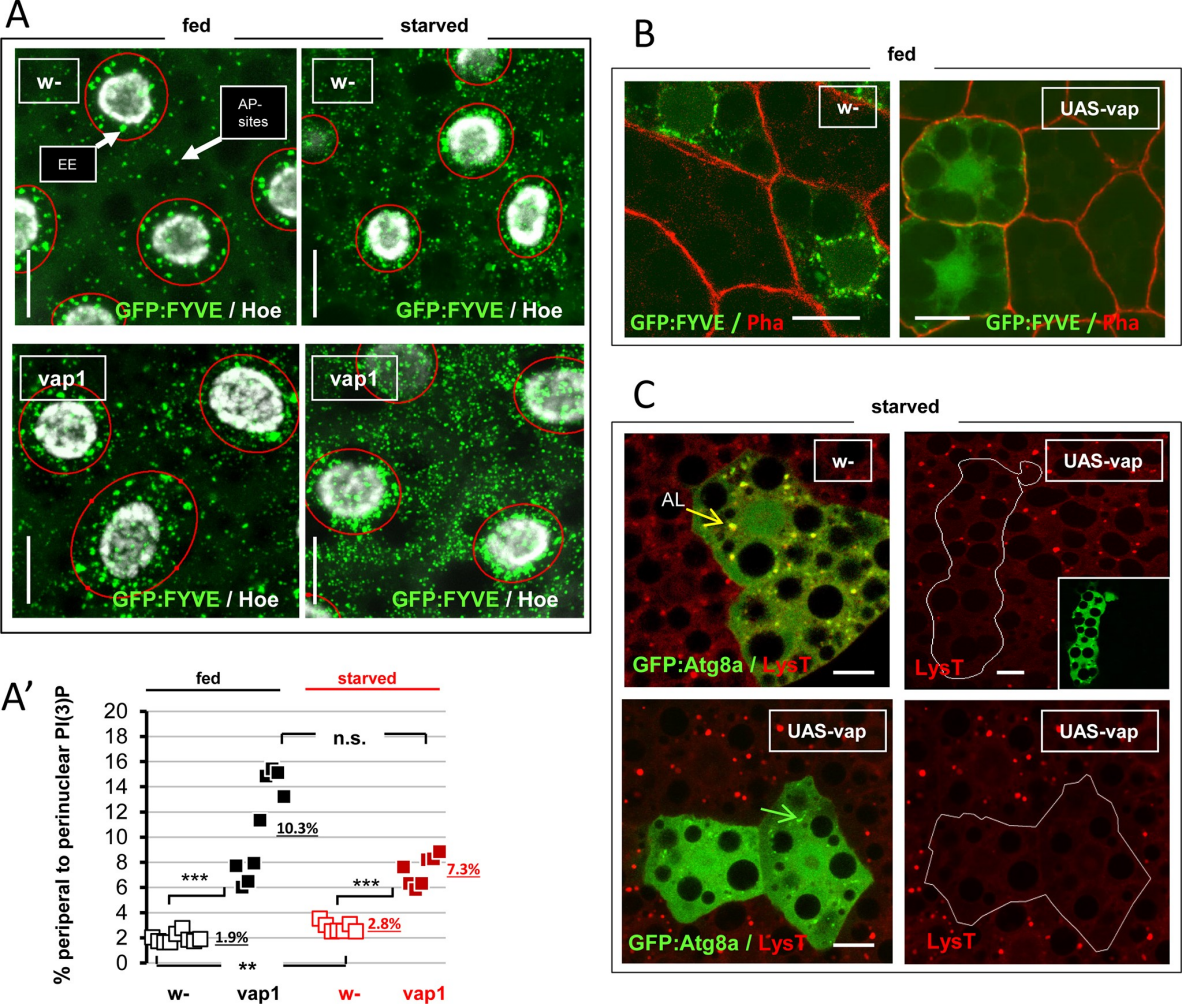
Loss of *vap* disturbs PI(3)P homeostasis in fed and starved cells. (A) *Cg-Gal4*-driven expression of the GFP:FYVE biosensor was performed in control, *w-* and mutant, *vap*^*1*^ fat cells, and images recorded from stage-matched early/ mid-3^rd^ instar live tissues form fed or 3h-starved animals. PI(3)P specific sub-pools comprising perinuclear early-endosomes (EE) and cytoplasmic-dispersed autophagy sites (AP sites), were delimited by red rings used for quantification following a setup described in [34] and **S2C Fig,** and graphed in details in **S1B Fig.** As noticed previously [34], starvation for 3h caused an elevation of the GFP:FYVE staining of both PI(3)P sub-pools, as found here in control, *w-* cells and in mutant, *vap*^*1*^ cells. Scale bars = 20 μm. (A’) Relative changes of cytoplasmic vs perinuclear PI(3)P were scored here. The respective GFP+ areas in selected acquisitions encompassing 3–4 cells and 7–9 depth sections each (corrected for nuclei numbers), were plotted. Underlined numbers are the mean relative percentage of cytoplasmic PI(3)P fractions in indicated categories. Control, *w-* fed: 1.9% (+/- 0.37) and starved: 2.8% (+/- 0.40). Mutant *vap*^*1*^ fed: 10.3% (+/- 3.9) and starved: 7.3% (+/- 1.2). The cytoplasmic PI(3)P fractions of *vap*^*1*^ fed and starved cells is significantly increased compared to controls: Fed, *vap*^*1*^ vs control, *w-* = 5 fold increase (p<0.0001). Starved, *vap*^*1*^ vs control, *w-* = 3 fold increase (p<0.001). In controls, starvation produced low but significant elevation of the relative cytoplasmic PI(3)P fraction (2.8% vs 1,9%; p<0.002). No starvation mediated elevation or clear decline is observed in *vap*^*1*^ cells (7,3% vs 10,3%; p>0.05). Note the dispersion of values in mutants as often the case. Significances are from Student’s *t*-tests. (B) Clonal expression of an *UAS-vap* wild-type transgene in fed larval fat body cells in the presence of the GFP:FYVE biosensor was induced using the *Act>CD2>Gal4* flipout cassette method. When compared to control, *w-* clones, clonal excess of Vap produced a complete absence of perinuclear GFP with rare remaining PI(3)P spots at the cell periphery. Scale bars = 20 μm. (C) Clonal expression of an *UAS-vap(wt)* transgene in the presence of the autophagosome marker *GFP*:*Atg8a* in larval fat cells was obtained as above, but larvae were starved for 3h and cells were additionally stained with LysoTracker red. Clones with excess Vap (inset and delimited by white lines) shows fewer or not any stained lysosomes (Top and bottom right panels respectively) compared to the wild-type neighboring cells, whereas GFP:Atg8a-labeled autophagomes is reduced to tiny GFP:Atg8a-positive structures (green arrow) as compared to starvation-induced autolysosomes (AL, yellow arrow) in control, *w-* clones. Scale bars = 20 μm. Genotypes. (A) Control: *w*^*1118*^*/Y; cg-GAL4/ UAS-GFP*:*myc*:*2xFYVE*. Assay: *vap*^*1*^*/Y; cg-GAL4/ UAS-GFP*:*myc*:*2xFYVE*. (B) Control: *w*^*1118*^*/ hsFLP*^*12*^*; UAS-GFP*:*myc*:*2xFYVE*, *Act>CD2>GAL4/+*. Assay: *w*^*1118*^*/ hsFLP*^*12*^*; UAS-Vap*:*myc*^*16*.*4*^*/+; UAS-GFP*:*myc*:*2xFYVE*, *Act>CD2>GAL4/+*. (C) Control: *w*^*1118*^*/ hsFLP*^*12*^*; Act>CD2>GAL4*, *UAS-GFP*:*Atg8a/+*. Assay: *w*^*1118*^*/ hsFLP*^*12*^*; UAS-Vap*:*myc*^*16*.*4*^*/+; Act>CD2>GAL4*, *UAS-GFP*:*Atg8a/+*.

To search for more indications on changed basal autophagy status in the *vap* fat bodies, we looked to the distribution of cellular PI(3)P, using the GFP:FYVE biosensor to reveal nucleating autophagy membranes sites ([34, 60] and Materials and Methods). In normal fat cells, PI(3)P-specific labeling comprises perinuclear, Rab5-containing, early endosomes as well as a cytoplasmic-dispersed labeling, which depicts nascent, *Atg8a*-positive, autophagosomes ([34] and **S2C Fig**). Interestingly, *vap*^*1*^ cells showed elevated distribution of the GFP:FYVE signals compared to control cells in both fed and starved animals (**Fig 2A**). Furthermore, early endosome sites and incipient autophagosome sites were affected (**S1B Fig** for quantification and **S2D Fig** for endosome-specific tracer incorporation). When the fraction of peripheral versus perinuclear fluorescence was expressed, fed *vap* cells scored a mean of 10.3% compared to 1.9% in control cells, and this was not heavily changed in cells of starving animals (*vap*^*1*^: 7.3%, control: 2.8%) (**Fig 2A’**). That elevated PI(3)P level is a feature of *vap* animals was confirmed biochemically, using specific ELISA assays reporting PI(3)P content in total lipid extracts of mid-3^rd^ instar larvae (**S1C Fig**). These observations indicated that in the absence of *vap*, homeostatic levels of PI(3)P were upregulated altogether, along with an elevation of the pool of PI(3)P assigned to autophagosome biogenesis.

To understand further the relation between *vap* and the generation of PI(3)P in fat cells, we next over-expressed wild-type Vap protein into them. In fed animals, this eliminated most of the PI(3)P labeling (**Fig 2B**). Interestingly, excessive Vap also inhibited lysosome and autophagosome inductions in the starved fat cells when labeled with LysoTracker and *GFP*:*Atg8a*/LC3 respectively (**Fig 2C**). These last effects are near phenocopies of the loss of *Vps34* in identical conditions [34]. Indeed this enzyme serves as the major pathway for PI(3)P formation in this cell type [34]. We accordingly found the reverse effect, that clonal expression of excessive *Vps34* phenocopied *vap* mutant effect of PI(3)P expansion (**S2A Fig**). Finally we also verified that dominant-negative *Vps34* expression abrogated GFP:FYVE labeling in both control and the *vap*^*1*^ mutant context (**S2B Fig**).

Consistent with Vps34 being an important promoter of autophagy [34, 35, 61], we conclude that *vap* likely controls basal autophagy function by oppositely acting to the Vps34 lipid kinase or PI(3)P formation. These activities in turn, are impinging on the rate of basal autophagy.

### II. Vap-dependent control of basal autophagy alters fat body cell growth

**Fig 3:**
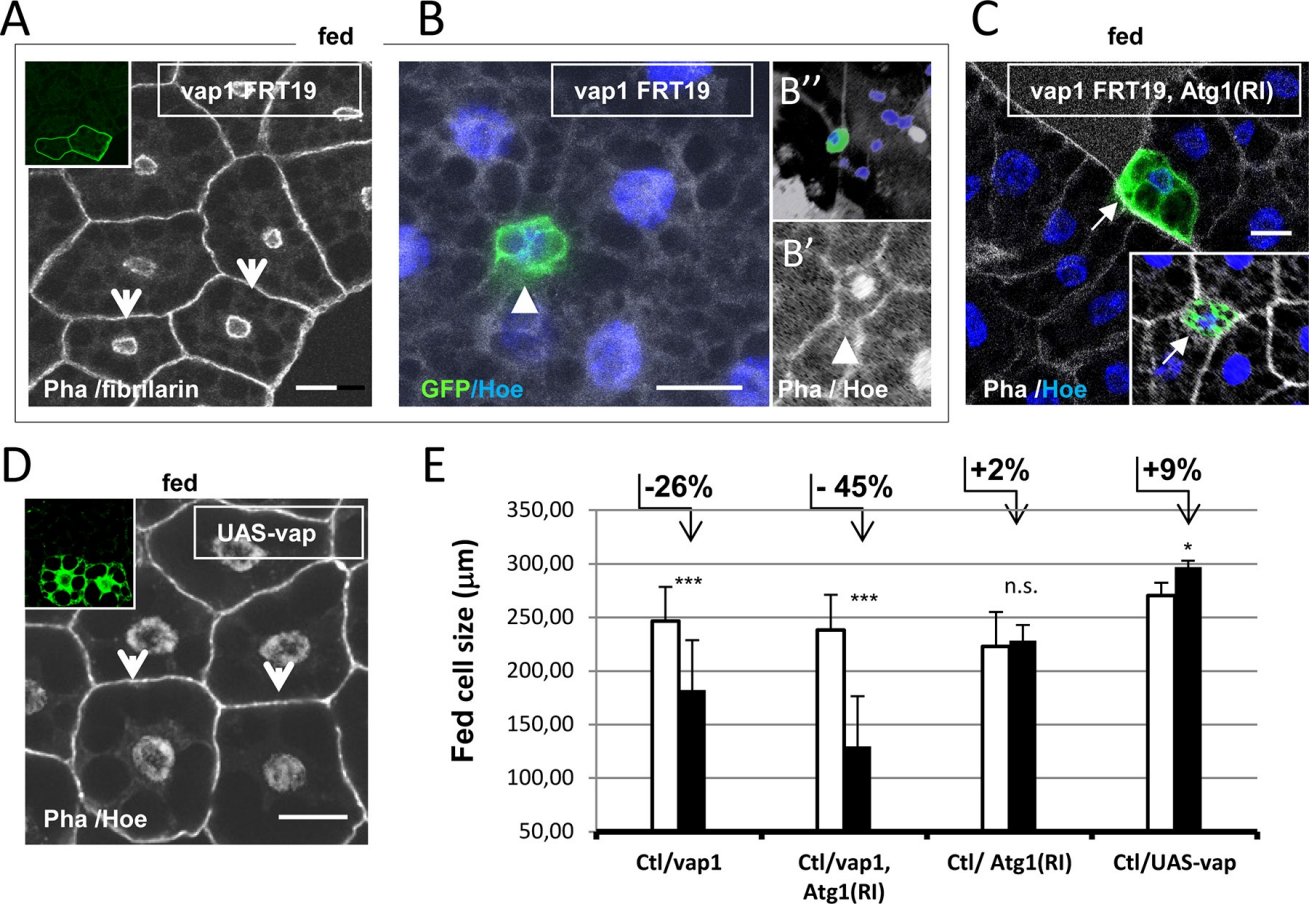
Fat cell clones of manipulated *vap* activity showed growth competitive phenotypes. (A) Clonal loss of *vap* in fat bodies of well-fed animals was generated by the MARM, GFP-positive labeling technique and analyzed in mid-3^rd^ larvae. Clonal *vap*^*1*^ cells (inset and arrows) grown for ca. 88h, shows autonomous cell size reduction compared to wild-type neighboring cells used as controls (Ctl). (B) An extreme case of disfavored growth of a *vap*^*1*^ mutant clone in the process of active elimination (size diminished by 60% and corresponding reduction of nucleus size). (B’) Mutant cell lost part of the phalloïdin-labeled actin cytoskeleton at a cellular contact (arrowhead). (B‘‘) A similar clone in the process of nuclear fragmentation is extruded from the tissue. (C) Doubly mutant clones of *vap*^*1*^ and *tubulin-Gal4* driven *Atg1(RI)* cells, were generated in fed animals and analyzed as in A (inset: a distinct clone). If anything, these shows enhanced cell size reduction rather than suppression of cell growth. Such a synergism could be related to the autophagy-independent requirement of Drosophila *Atg1* [43]. *Atg1(RI)* expression alone in these conditions has not detectable effects (E). See validation of experimental setting and used *Atg1(RI)* construct in **S3A Fig**. (D) Clonal expression of an *UAS-vap(wt)* transgene in larval fed fat body cells was achieved using the *Act>CD2>Gal4* flipout cassette method. A mild and autonomous increase in cell size is observed. Scale bars in all panels = 20 μm. (E) Relative cell-size changes of manipulated cell clones in A-D were quantified. Lengths of the cell contours (in μm) were determined from images of the clones and compared to wild-type cells contours in the same images. MARCM and flipout genetic setting resulted in normal sized GFP-positive cells. (Clt n = 23 /*vap*^*1*^ n = 13; Ctl n = 14 /*vap*^*1*^, *Atg1(RI*) n = 10; Ctl n = 8 /*Atg1(RI)* n = 8; Ctl n = 10 /*UAS-vap* n = 12). Error bars are mean differences; significances are from Student’s *t*-tests Genotypes. (A-B”) *vap*^*1*^, *FRT19A / tub-GAL80*, *hsFLP1*, *FRT19A; UAS-CD8*:*GFP/+; tub-GAL4/+*. (C, E) Control: *FRT19A / tub-GAL80*, *hsFLP1*, *FRT19A; UAS-CD8*:*GFP/+; tub-GAL4/ UAS-Atg1(RI)*. Assay: *vap*^*1*^, *FRT19A / tub-GAL80*, *hsFLP1*, *FRT19A; UAS-CD8*:*GFP/+; tub-GAL4/ UAS-Atg1(RI)*. (D) Assay: *w*^*1118*^*/ hsFLP*^*12*^*; UAS-Vap*:*myc*^*16*.*4*^*/+; Act>CD2>GAL4*, *UAS-GFP/ +*.

We then asked if the decreased fitness of *vap* mutant organisms could be linked to the changing basal autophagy. To challenge the fitness capacity of *vap* mutant at a cellular level, we generated mutant fat body cell clones in well fed animals, tracking GFP-marked mutant cells (**Fig 3A and 3B**). *Vap*^*1*^ clones were rare and autonomously differentiated as small-sized cells with a mean size reduction of 26%, suggesting that mutant conferred reduced growth capacities, leading to eventual out-competition by wild-type neighboring cells ***(*Fig 3A and 3E**). Indeed, occasional *vap*^*1*^ cells were found eliminated as apoptotic cells, showing nuclear fragmentation and reduced actin at cell-cell contacts (**Fig 3B–3B”**). The size reduction of mutant *vap*^*1*^ cells, was not much changed in nutrient restricted conditions as compared to appropriate control (**S3A Fig**).

Similar cell undergrowth and elimination features has been observed when overabundant autophagy was induced following ectopic expression of the Atg1 kinase in fat cells of fed animals (i.e. in absence of starvation signaling [14]). This treatment however, led to greater cell growth inhibition with a mean size reduction of 94% and many clones engaging apoptosis [14]. To investigate whether the small-sized *vap* clones could be resulting in a neo-activation of *Atg1*, we studied cells that lost *vap* and were simultaneously silenced for *Atg1* by RNAi-mediated expression. Doubly mutant clones failed to rescue any size reduction, whereas single *Atg1(RI)* clones were iniquitous in these conditions (**Fig 3C and 3E and S3A Fig**). This indicates that the reduction in size of the *vap* cells is an *Atg1*-independent effect. To complement the loss-of-function studies we then asked if Vap overexpression could likewise affect the growth of fed fat cells. Indeed, clonal excess of Vap resulted in a 9**%** increase in relative cell size (**Fig 3D and 3E**) indicating that the gain of *vap* function conferred a weak growth advantage. In this case, a beneficial cell-energetical balance could be resulting in a diminution of endogenous (standard) basal autophagy rate. We conclude that normal *vap* activity contributes to balanced fitness characteristics of the growing fat cells by ensuring the most cost-effective basal autophagy rate.

### III. Starvation-induced autophagy is arrested in *vap* mutants

**Fig 4:**
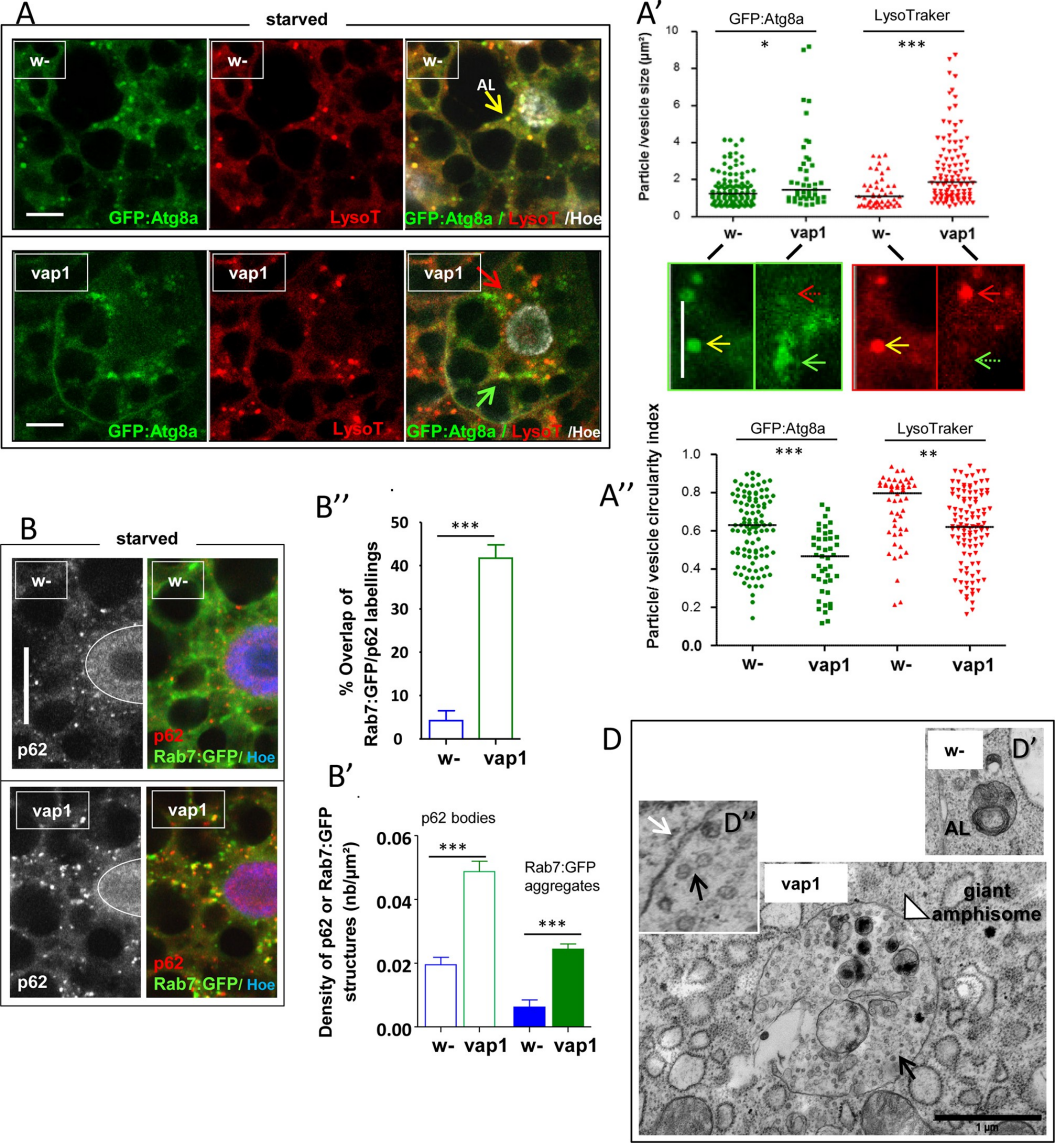
Loss of *vap* alters starvation-induced autophagy-membrane biogenesis. (A) The autophagosome marker GFP:Atg8a was expressed in control, *w-* or *vap*^*1*^ fat bodies using a *cg-Gal4* driver, and mid-3^rd^ instar larvae were starved for 1h30’. Fat bodies were dissected and stained LysoTracker red for an immediate observation of live tissue. Shown, are comparable images of control, *w-* and mutant, *vap*^*1*^ cells, which respectively scored 14 yellow vesicles depicting fused autolysosomes (AL) out of 52 total vesicles, and 1 yellow vesicle out of 51 total vesicles, indicating that no or rare autolysosomes had formed in the mutant. LysoTracker red signal area intersecting GFP:Atg8a signal area was 77,3% in control and only 2,3% in *vap*^*1*^ confirming the lack of fused structures. Scale bars = 10 μm. (A’, A”) Green and red vesicular-membrane signals were extracted from similar images and their size (in μm^2^) and circularity index (1.0 for perfect circles; 0.0 for elongated polygons) graphed as scatter plots (*w-*: n = 106 green vesicles, n = 49 red vesicles; *vap*^*1*^: n = 43 green vesicles, n = 107 red vesicles). Below are enlarged pictures of green and red single channel images for *w-* and *vap*^*1*^ respectively. Yellow arrows point to autolysosomes in *w-*. Green and red arrows point to autophagosome membranes and lysosome respectively, in *vap*^*1*^. Dotted arrows emphasize the absence of vesicular fusion. Scale bar = 10 μm. In A’, control, *w-* green and red vesicles has relatively packed distribution as a fraction of them derive from same autolysosomes. Mutant *vap*^*1*^ cells has non-fused green and red membranes (or vesicles) which are of larger sizes and wider distributions (green vesicles Mdn = 1,46 μm^2^ in *vap1* vs 1,25 μm^2^ in controls; red vesicles Mdn = 1,86 μm^2^ in *vap1* vs 1,10 μm^2^ in controls). These differences correlate with a decreased circularity index of the mutant particles in A” (green vesicles Mdn = 0,47 in *vap*^*1*^ vs 0,63 in controls; red vesicles Mdn = 0,62 in *vap*^*1*^ vs 0,79 in controls), indicating that mutant autophagosomes and lysosome membranes were wider and of uneven shapes. Medians are drawn as lines; significances are from Mann Whitney test. (B) The late endosome *Rab7*:*GFP* marker was expressed in control, *w-* and mutant, *vap*^*1*^ larval fat bodies using a *cg-Gal4* driver and 3h-starved fat cells were analyzed. Endogenous p62/SQSTM1 flux marker was detected by immunostaining. In control, *w-* starved cells, fine p62 bodies (grey or red) are detected over the cytosol whereas Rab7:GFP aggregates are forming independently of them. In *vap*^*1*^ cells both the density (B’) and size range of p62 bodies and Rab7:GFP aggregates (**S1D and S1D’ Fig**) are increased and the two markers match frequently (B”), suggesting accumulation of unresolved maturation intermediates in the mutant. Scale bar = 20 μm. (B’) The densities for p62 bodies and Rab7:GFP aggregates were compared in a selections of fat body cells samples of defined areas of control and mutant cell in B (*w-* n = 8, *vap*^*1*^ n = 13). Error bars are standard errors; significances are from ANOVA. (B”) The overlap between Rab7:GFP and p62 staining was determined in the set of cells used in B’. Rab7:GFP signal areas intersecting p62-positive pixels were expressed relative to total p62 staining areas. Significant intersections are only found in the case of starved *vap*^*1*^ cells. Error bars are standard errors; significances is from Student’s *t*-tests. (D-D”) TEM semi-quantitative analysis of autophagy structures found in fat body cells of 2h-starved early/mid-3^rd^ instar of control, *w-* and *vap*^*1*^ mutant (Table C in **S5 Fig**). Control, *w-* shows typical degradative autolysosomes (AL in D’; scale as in mutant below) of ca. 0.5 μm in size (28 cases of AL out of 41 scored autophagy-related structures). No autolysosomes were detected in *vap*^*1*^ samples. Instead, large hybrid organelles (arrowhead: giant amphisome) of greater than 2 μm are observed (14 giant amphisomes cases out of 38 scored autophagy-related structures). These have single-bilayered membranes (D” inset: white arrow) and are filled with intraluminal, electron-clear vesicles (black arrow) akin those of MVB (multivesicular bodies). These structures appear to match the accumulated maturation intermediate detected in B. Scale bar = 1 μm. Genotypes. (A) Control: *w*^*1118*^*/Y; cg-GAL4/ UAS-GFP*:*Atg8a/+*. Assay: *vap*^*1*^*/Y; cg-GAL4/ UAS- GFP*:*Atg8a/+*. (B, C, C’) Control: *w*^*1118*^*/Y; cg-GAL4/ UAS-Rab7*:*GFP/+*. Assay: *vap*^*1*^*/Y; cg-GAL4/ UAS-Rab7*:*GFP/+*. (D) Control: *w*^*1118*^*/Y*. Assay: *vap*^*1*^*/Y*.

To investigate the origin of the reinforced lysosomal-induction response observed in the starved *vap* animals and check if an increased basal autophagy rate would modify the implementation of stimulated autophagy, we compared the biogenesis of the starvation-induced autophagy between control and mutant *vap*^*1*^ fat bodies. Fusion into autolysosomes was assessed by combining GFP:Atg8a marker and LysoTracker dye to label autophagosome membranes and acidified lysosome structures, respectively. When controls were subjected to a short starvation period of 1h30’, we often saw fused autolysosomes structures forming yellow vesicles. (**Fig 4A**). On the contrary, *vap*^*1*^ mutant cells displayed broaden thought misshaped autophagosomes membranes (in green) next to enlarged lysosomes (in red; **Fig 4A and 4A’**). This suggests that fusion into degradation-active autolysosomes is not taking place in the mutant.

To explore further the state of starvation-induced autophagic flux in the mutant context we then analyzed the clearance of p62 /SQSTM1, which is involved in polyubiquitinated protein cargo-reception while bound to the internal autophagosome membranes [58, 62]. Because active autophagy led to degradation of p62 together with cargos [62], labeling of these structure is being revealed as fine cytosolic bodies due to natural protein turnover. Whilst this is observed in the starved control, *w-* cells (**Fig 4B**), p62 bodies of *vap*^*1*^ cells were more abundant and had an increased size, indicating its accumulation (**Fig 4B and 4B’ and S1D Fig**). In the same experiment, we used Rab7:GFP marker to visualize late endosome and late autophagosome compartments (**Fig 4B**). In control starved cells, the Rab7:GFP marker are forming aggregated concentrations over the cytosol that never matched the p62 particles. However, the two markers showed considerable overlap in the *vap* context (**Fig 4B”**). The congruence of Rab7 and p62 that both accumulated in starved *vap* cells (**Fig 4B’**) is consistent with a block of autophagic flux at late maturation stage [63].

Electron microscopy analysis (TEM) of samples of starved control and *vap* animals confirmed the lack of typical autolysosomes structures in the mutant cells. However, these were replaced by stalling hybrid-intermediate structures with characteristics of giant amphisomes (**Fig 4D–4D” and S5C Fig**).

Together, our observations revealed an expansion of autophagy-related endomembranes and an accumulation of enlarged maturation intermediates, consistent with elevated levels of PI(3)P promoting excessive membrane influx and fusions [64]. With these data in mind, we conclude that the arrested autophagy maturation seen in starved *vap* cells is a consequence of disrupted PI(3)P-dependent membrane dynamics [65].

### IV. Concurrent defects in basal and starvation-induced autophagy led to starvation hypersensitivity of *vap* adult flies

**Fig 5:**
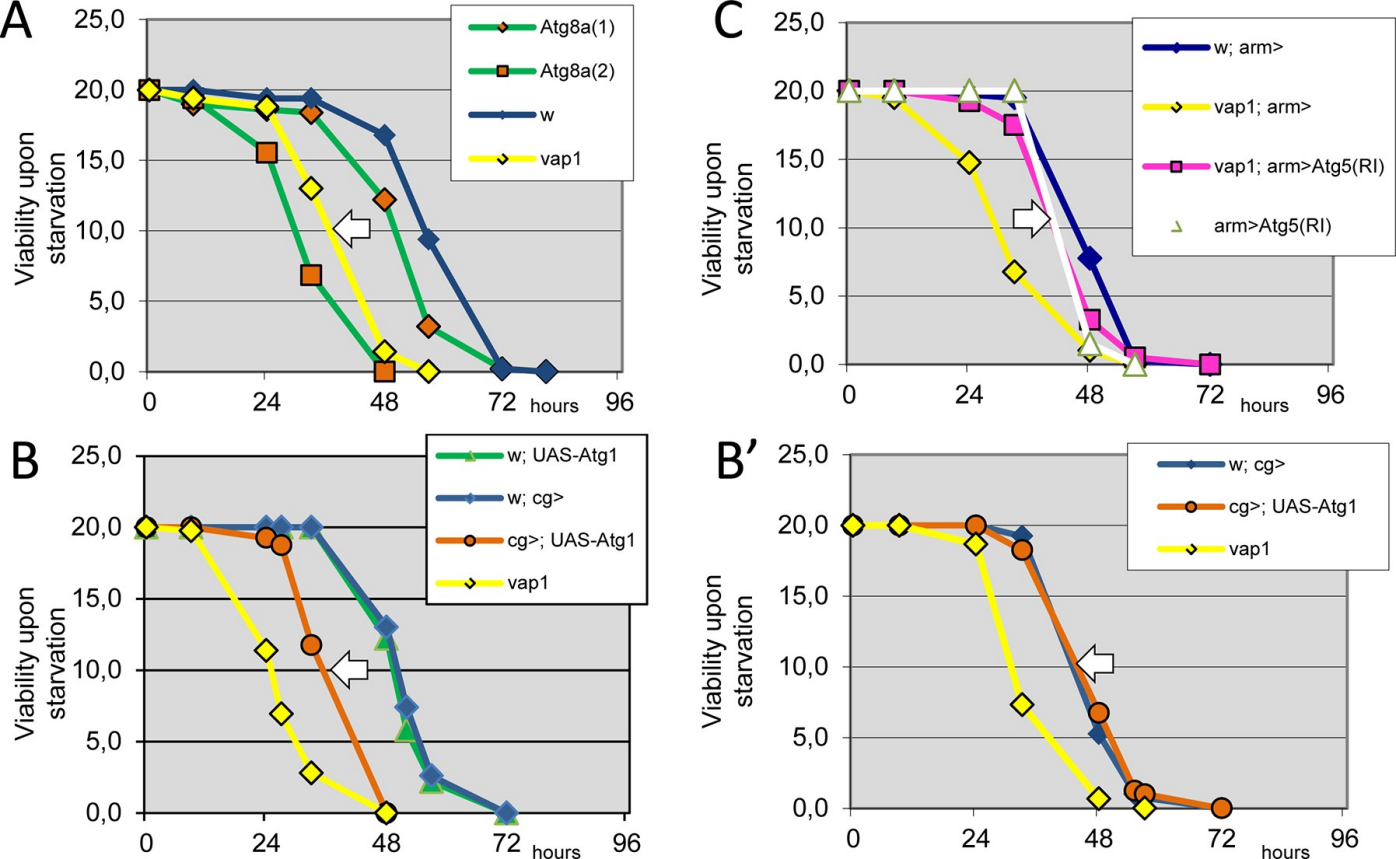
Starvation-sensitivity assays define the range of autophagy defects in *vap* flies. The survival rate of 3 day-old adult males of indicated genotypes was recorded at 25°C in condition of complete food deprivation (see **S4A–S4C Fig** for initial characterization). (A) The *vap*-dependent starvation sensitivity (white arrow) was compared to weak (*Atg8a*^*1*^) and strong (*Agt8a*^*2*^) alleles of *Atg8*a. *Atg8a*^2^ flies showed slightly altered development that might contribute to its greater sensitivity to starvation. (B-B’) Starvation sensitivity effect, as assayed at 25°C, is partially recapitulated by flies that were ectopically expressing an *UAS-myc*:*Atg1* transgene (Materials and Methods) along fat cell development performed at 25°C (white arrow in B) when driven by *cg-Gal4*. As a control, there is no detectable starvation sensitivity (as assayed at 25°C) using identical flies (*UAS-myc*:*Atg1* /*cg-Gal4*) that developed at 18°C to minimized transgene expression (white arrow in B’). Ectopic expression of *Atg1* during development is therefore responsible for the sensitivity effect found in B. (C) The *vap*-dependent starvation sensitivity is suppressed (white arrow) by co-expressed *Atg5(RI)* using the broadly expressed *arm-Gal4* driver. Genotypes. (A) Control: *w*^*1118*^*/Y*. Assay *vap*^*1*^*/Y*. *Atg8a*^*1*^*/Y*. *Atg8a*^*2*^*/Y*. (B, B’) Control: *UAS-myc*:*Atg1/+* and *vap*^*1*^*/Y* and *vap*^*1*^*/Y; cg-GAL4/+*. Assay: *vap*^*1*^*/Y*. *cg-GAL4/ UAS-myc*:*Atg1(RI)/+*. (C) Control: *arm-GAL4/+* and *vap*^*1*^*/Y; arm-GAL4/+* and *arm-GAL4/ UAS-Atg5(RI)/+*. Assay: *vap*^*1*^*/Y; arm-GAL4/ UAS-Atg5(RI)/+*.

We next considered the fallout of the discovered autophagy dysregulations in *vap* mutants. We noticed that *vap* flies were quite hypersensitive to acute food deprivation as they would not survive for long without providing a simple source of energy such as carbohydrate (**S4A and S4C Fig**). This sensitivity was apparently not a consequence of the aged-brain defect found in older *vap* adults (**S4B Fig**). We then compared the relative resistance to starvation of *vap* flies to wild type and to *Atg8a* mutant strains. Like *vap*, *Atg8a* mutants were mostly viable as adults despite their defect in starvation-induced autophagy preventing substantial production of recycled nutrients [66]. Consistent with such paradigm [43], *vap* flies displayed midway level of sensitivity to starvation when compared to *Atg8a*^*1*^ hypomorphs and to the sub-viable *Atg8a*^*2*^-null mutants (**Fig 5A**).

We then assessed intra GAP gene sub-family rescue and tissue-specific rescue of *vap* function, using transgenes encoding a wild-type Vap protein or the related wild-type NF1 and GAP1 GAP-proteins. Complementation of *vap*-specific sensitivity to starvation was only successful using the Vap transgene (**S4D and S4F Fig**). Further, by using Gal4 drivers expressed in defined tissues (i.e. fat body, muscles or nerve cells), the rescue varied from partial to complete rescue. In one case, the rescue caused resistance to starvation greater than control (**S4E–S4H Fig)**. These experiments, however, could not establish if rescue accounted for complementation of the starvation-induced defects or the basal autophagy defect of *vap*, or the two simultaneously. We reasoned that resistance to starvation depends on the amount and quality of nutrient stores, as well as on the capacity to remobilize those stores. Because we found that *vap* flies had lower mass and lower TAG, we hypothesized that elevation of basal-autophagy rate during the course of animal growth might indeed lead to starvation sensitivity of the adult flies. To test this, we expressed low levels of ATG1 in the fat tissue [55] and challenged the resulting adults for resistance to acute starvation. Indeed, *Atg1*-expressing flies led to a sensitivity ranging 2/3 that of *vap* flies (**Fig 5B and 5B’**). Finally, we found that *vap*-dependent sensitivity to starvation was alleviated by general silencing of *Atg5*, following RNAi expression (**Fig 5C**). We conclude that the starvation-sensitivity response (and diminished mass and nutrient stores) of adult *vap* flies could only be explained when defects in both remobilization and basal autophagy are taken into consideration.

### V. Sprint is essential for vap activity and autophagy membrane inflation

Having described a range of phenotypes pointing to deregulated autophagy, we inquired about the potential mechanisms that could sustain this function for *vap*. As a GAP regulator, we thought that *vap* acted via intermediary GTPase. However, by performing structure-function analysis using a PI(3)P inhibition assay in fat cell, we found that Vap activity could be uncoupled of its GTPase binding function (**S5A Fig**). Instead, the analysis pointed to a requirement for two SH2 domains of its NH2-terminal region. The same domains were previously found to bind a range of Tyr-phosphorylated partners of Vap proteins, of which the guanine nucleotide exchange factor (GEF) Sprint appeared a major one [48]. Indeed phospho-Spri proteins were binding to Vap *in-vitro* and *in-vivo* through its N-terminal SH2 domains [48].

To check if *spri* could be contributing to autophagy in any fashion, we analyzed *spri-*silenced fat body clones, using starvation-induced autophagy as readout for vesicular endomembrane biogenesis.

**Fig 6:**
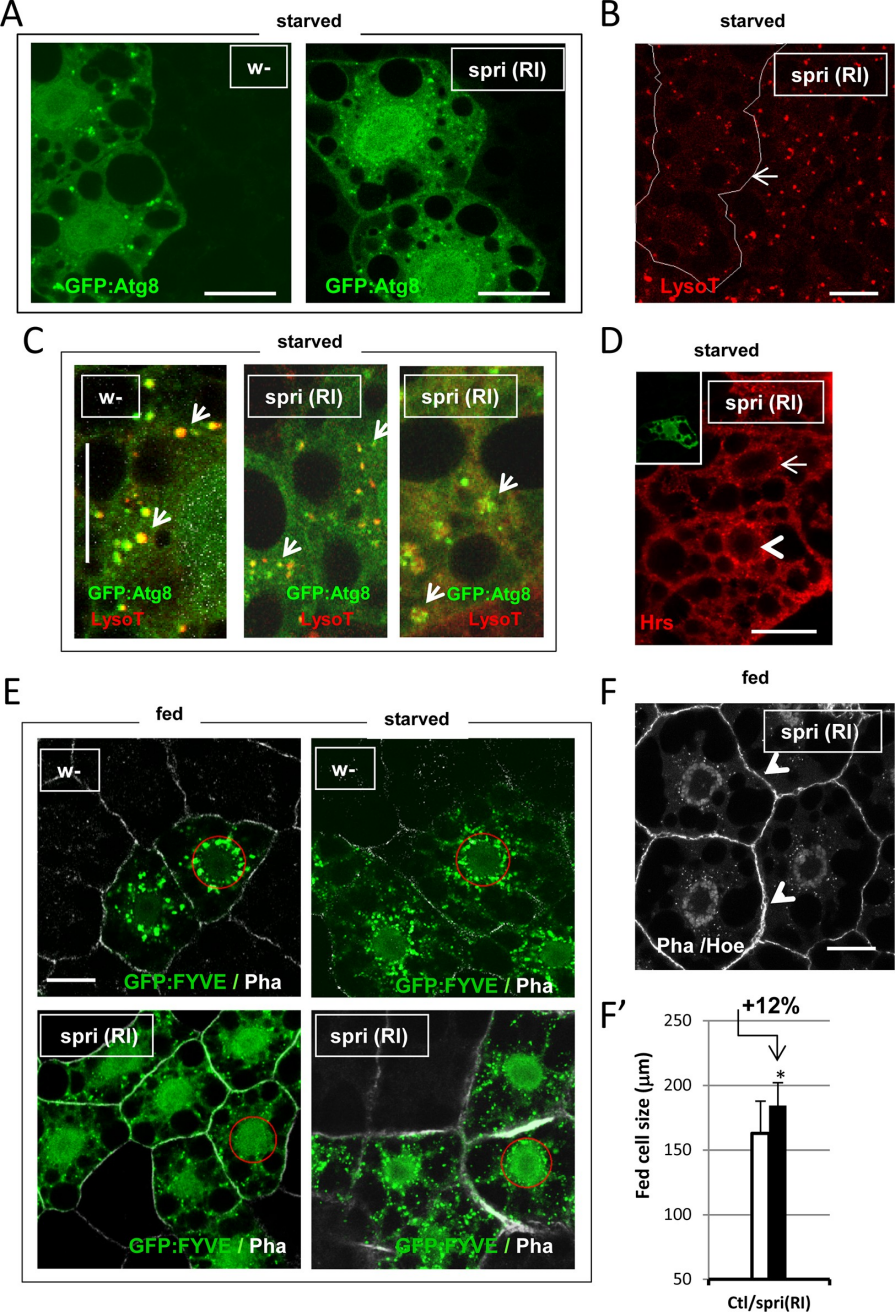
Sprint appears to be dispensable for starvation-induced autophagy. (A) Clones of *spri(RI)* fat cell or control, *w-* were generated together with GFP:Atg8a autophagosome marker expression, using the *Act>CD2>Gal4* flipout cassette method. Larvae were starved for 3h, and fat body stained with LysoTracker red when needed. *spri* mutant cells shows autophagosomes of reduced size compared to that of control, *w-* cells. (B) *spri(RI)* clonal cells (delimited by a white line) shows small-sized or tiny lysosomes as stained with LysoTracker red. (C) A close view of autophagy vesicles shows fused ‘yellow’ autolysosomes (arrows) in control, *w-*whereas mutant *spri(RI)* forms small but fused autolysosomes (arrows) and more sporadically, none-fused but tethered autophagosome and lysosome vesicles (arrows and **S1 Table**). (D) *spri(RI)* clonal cells were stained for the ESCRT-0 early endosome marker Hrs, showing perinuclear accumulation of these structures (arrowhead) as compared to control nearby cells (arrow). This either resulted from blocked progression into endosomal MVB (as intraluminal vesicles formation of MVB requires PI(3)P for scission from the surface [53]), or else from ineffective maturating fusion of autophagosome to the MVB [78]. (E) Clones of *spri(RI)* fat cell or control, *w-* were generated together with the GFP:FYVE biosensor, using the *Act>CD2>Gal4* flipout cassette method. Fed or 3h-starved early/mid-3^rd^ instar larvae were analyzed in fixed tissues. Fed *spri*-silenced cells form fine PI(3)P foci at the periphery and the perinuclear pools of PI(3)P is reduced compared to control (red circles for delimitation of the pools, see **S2C Fig**). Free GFP:FYVE fluorescent probe remains in the cytosol and nucleus as in the case of *UAS-vap* wild-type transgene expression (**Fig 2B**). Upon starvation, *spri*-silenced cells partly recovered from these defects, including the formation of new perinuclear PI(3)P pools. The phenotype is subjected to variation (**S1 Table).** A detailed quantification is found in **S5B Fig**. (F, F’) Clonal *spri(RI*) cells developed in normally fed animal has mild increased size. Cell size was quantified relative to neighboring control cells as in **Fig 3.** (Clt n = 24; *spri-* n = 21). Error bars are mean differences; significance is from Student’s *t*-tests. Scale bars in all panels = 20 μm. Genotypes. (A-D, F, F’) Control: *w*^*1118*^*/ hsFLP*^*12*^*; +/+; Act>CD2>GAL4*, *UAS-GFP*:*Atg8a/+*. Assay: *w*^*1118*^*/ hsFLP*^*12*^*; UAS-spri(RI)/ +; Act>CD2>GAL4*, *UAS-GFP*:*Atg8a/+*. (E) Control: *w*^*1118*^*/ hsFLP*^*12*^*; UAS-GFP*:*myc*:*2xFYVE*, *Act>CD2>GAL4/+*. Assay: *w*^*1118*^*/ hsFLP*^*12*^*; UAS-spri(RI)/ +; UAS-GFP*:*myc*:*2xFYVE*, *Act>CD2>GAL4/+*.

Strikingly, *spri* mutant cells showed a reduced aspect of both type of autophagy vesicles, i.e. GFP-labeled autophagosomes and LysoTracker-labeled lysosomes (**Fig 6A and 6B**). A fraction of *spri-*silenced cells showed aborted fusions into autolysosomes and some showed impaired endosomal trafficking as revealed by early endosomes, Hrs marker accumulation (**Fig 6C and 6D and S1 Table**). Both anomalies are indicative of defective vesicular fusion events (**Fig 6D**). Consistent, general PI(3)P labeling was diminished in both fed and starved mutant cells (**Fig 6E and S5B Fig**). Finally, we found that clonal *spri-*silenced cells had a mild growth advantage when generated in fed animals (**Fig 6F and 6F’**) similar to those clones in which Vap is overexpressed. Together, we conclude that the detected alterations of *spri-* cells materialized the reverse effects to that of *vap* mutant cells, that is larger clonal cell size and lowered PI(3)P-dependent endomembrane influx, leading to small autolysosomes or ineffective fusions of vesicles.

**Fig 7:**
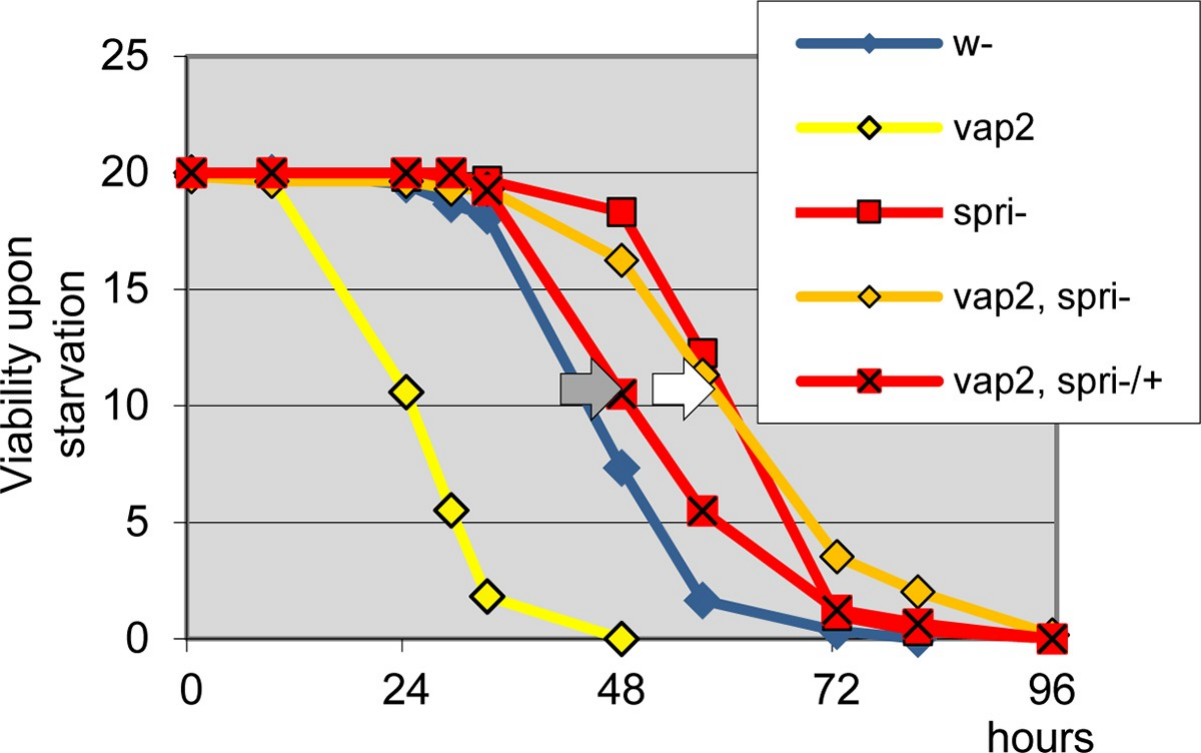
Sprint is essential for the starvation-sensitivity phenotype of *vap*. The genetic relationship between *vap* and *spri* mutants was evaluated using the starvation-sensitivity set up as in **Fig 5 and S4 Fig**. Females of indicated genotypes were used to record for survival upon complete nutritional deprivation at 25°C. The strong sensitivity to starvation of *vap*^*2*^ mutants is suppressed when combined to the *spri*^*6G1*^-null mutants (white arrow). Thus, Spri acts after Vap. When in the *vap* mutant context, *spri* heterozygous flies (*vap-/-*, *spri-/+*) shows midway suppression (grey arrow), and thus dosage effect of *spri+*. Single mutant *spri*^*6G1*^ or double mutant *vap*^*2*^, *spri*^*6G1*^ has greater resistance than control. Male genotypes resulted in all the same effects. Genotypes. Control females: *w*^*1118*^*/ w*^*1118*^. *vap*^*2*^*/ vap*^*2*^. *spri*^*6G1*^*/spri*^*6G1*^. Assay females: *vap*^*2*^, *spri*^*6G1*^*/ vap*^*2*^, *spri*^*6G1*^. *vap*^*2*^, *spri*^*6G1*^*/ vap*^*2*^, *+*.

To establish a functional link between Spri and Vap, we asked if *spri* mutant modified the sensitivity to starvation of *vap* using double mutant flies. Indeed, *spri*^*6G1*^-null mutants fully suppressed the starvation sensitivity associated to *vap* (**Fig 7,** white arrow), indicating that *spri* is responsible for the deregulation observed in *vap*. Additionally, we found that *vap*-/-, *spri-/+* heterozygotes display midway suppression (grey arrow in **Fig 7**). This dosage effect shows that a single *spri+* dose can confer sensitivity to starvation when *vap* is absent. Altogether, this points to a role for Vap as a repressor of Spri activity.

Interestingly, *spri* mutants themselves were not sensitive to starvation. On the contrary, they showed greater resistance than controls (white arrow in **Fig 7**).This finding lead us to conclude that *spri* is not essential for starvation-induced autophagy. Instead, the slight starvation resistance of *spri* flies could result in a loss of normal endogenous basal autophagy.

In summary, Vap negatively controls Sprint activity to ensure proper inflation of PI(3)P-dependent endomembranes as they evolve into autophagy and endolysosomal vesicles. Genetically, *vap* had dual impact on autophagy, acting on the basal and the starvation-induced processes. However, *spri* might be essential for basal autophagy only.

### VI. The activities of both Vap and Sprint regulate endocytic Rab5 levels

**Fig 8:**
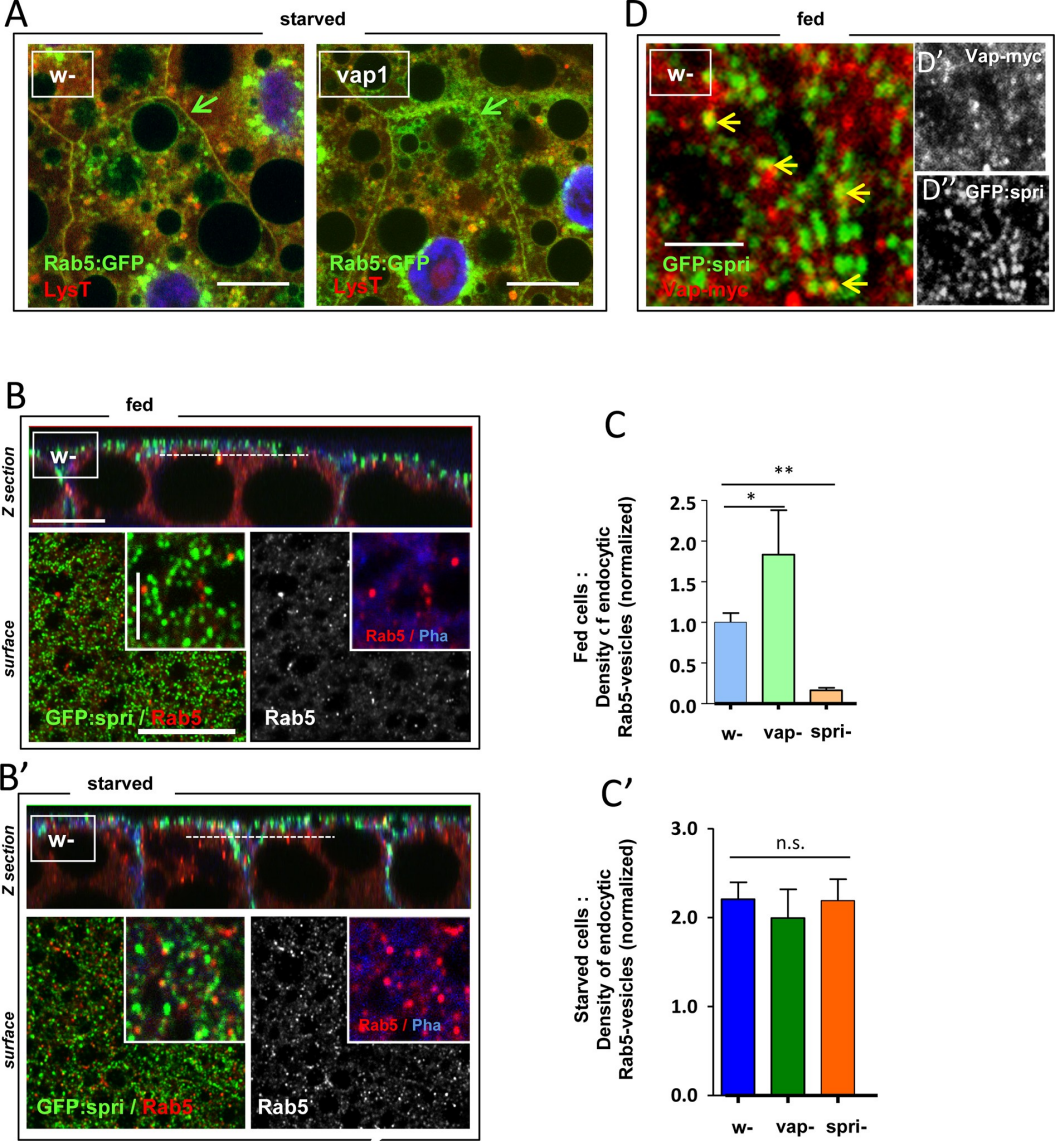
Regulation of Rab5-positive vesicle formation in the endocytic compartment of fat cells. (A) The Rab5:GFP tracer was expressed in control, *w-* or *vap*^*1*^ fat bodies using *cg-Gal4*. Mid-3^rd^ instar larvae were starved for 4h before staining of the fat tissue with LysoTracker and live cells analysis. Compared to control, *w-* cells, *vap*^*1*^ cells shows enhanced vesicular Rab5 trafficking near the cell membrane (green arrows). Scale bars = 20 μm. (B, B’) Cortical and endocytic compartment organization of fat body cells. Control strain, *w-* was used to express a *GFP*:*Spri* transgene using a *cg-Gal4* driver. Fed fat cells or 1h30’-starved cells of early-3^rd^ instar larvae were analyzed after immunostaining for Rab5, and F-actin (Pha) plus nuclei (Hoe) labeling. Each panel shows: (Top) Z-sections images of dome-shaped fat cells reconstituted from serial XY optical sections exemplified below. (Below left images) Colored surface plan views shows the distribution of GFP:Spri present as spots close to the plasma membrane. (Below right images) Underneath plan section view of Rab5 (grey scale or red) carried along the dotted lines shown in the Z section views. Scale bars in Z and plan sections = 10 μm. The GFP:Spri labeling is imbricate with the cortical F-actin stain (blue) above the vesicular Rab5 staining (Rab5 signal overlapped 2,6% of GFP:Spri signal). Insets: high magnification images. Scale bars = 2.5 μm. In B’, Rab5-positive vesicles are clearly increased after short starvation period (Rab5 signal overlapped 11.7% of GFP:Spri signal). Scale bars as in B. (D-D”) GFP:Spri and myc-tagged Vap were coexpressed from an *Act-Gal4* driver in fat cell clones of fed control, *w-* animals. A plan surface view reveals the GFP:Spri spots together with immunostained Vap:myc proteins in red (also shown in the respective gray scale images). Vap-specific staining overlapped 8% of GFP:Spri signal (yellow arrows) as determined in wide field images. Spri proteins were found engaged with several partners of the cell cortex [79] and only phospho-Tyrosylated Spri associated to Vap [48]. This may account for the relatively low coincidence of the two proteins. Scale bar = 2 μm. (C, C’) The density of endocytic Rab5-positive vesicles was quantified in control and mutant fat bodies. Experimental setting was exactly as in B, but used the genotype given below. Single plan images along the dotted line in the Z views were used to measure Rab5-positive vesicle densities (numbers of vesicles per μm^2^ of cellular area) as taken from wide field images. Values were normalized to the fed control, *w-; cg-Gal4/+*. Data using three different *GFP*:*Spri* transgenes were pooled. *spri*^*6G1*^-null cells were analyzed as above but the *GFP*:*Spri* transgene was omitted. In fed cells, the absence of *vap* causes in an elevation of ca. 2 fold of the Rab5-positive vesicle density, whereas the loss of *spri* results in a 6 fold reduction of vesicle density. Starvation is associated with a rise of 2.2 fold of vesicular Rab5 in control cells and this is not significantly different in the *vap* or *spri* mutant cells.(*w-* fed, n = 13; *vap*^*1*^ fed, n = 5; *spri*^*6G1*^ fed, n = 3; *w-* sta, n = 13; *vap*^*1*^ sta, n = 5; *spri*^*6G1*^ sta, n = 3). Error bars are standard errors; significances are from ANOVA. Genotypes. (A) Control: *w*^*1118*^*/Y; cg-GAL4/ UAS-Rab5*:*GFP/+*. Assay: *vap*^*1*^*/Y; cg-GAL4/ UAS-Rab5*:*GFP/+*. (B, B’) Assay: *w*^*1118*^*/Y; cg-GAL4/ UASp-GFP*:*Spri*^*9M*^. (C, C’) Control: *w*^*1118*^*/Y; cg-GAL4/+; UASp-GFP*^*7M or 8M or 9M*^*/+*. Assay: *vap*^*1*^*/Y; cg-GAL4/+; UASp-GFP*:*Spri*^*7M or 9M*^*/+*. *spri*^*6G1*^*/Y; cg-GAL4/+*. (D) Assay: *w*^*1118*^*/ hsFLP*^*12*^*; UAS- Vap*:*myc*^*16*.*4*^*/ UASp-GFP*:*Spri*^*9M*^*; Act>CD2>GAL4/+*.

Spri is reported both as regulator of endocytosis and as the ortholog of the RIN1-3 family of Ras activated Rab5 GEF that comprise Vps9-nucleotide exchange domains [47, 52]. We thus search for an articulation of the Vap/Spri modules to functional Rab5 in the fat body cell context. Using the Rab5:GFP tracer, we saw expanding GFP-labeled vesicular structures at, and near, the cell membrane of *vap*^*1*^ mutant cells after starvation was induced (**Fig 8A**).

We then mapped Spri and Vap protein as well as Rab5 molecules in this tissue. Using driven *GFP*:*Spri* expression we saw a spotty pattern of the Spri fusions at the plasma membrane (**Fig 8B**). This fluorescence partially colocalized with coexpressed tagged Vap (**Fig 8D**). The base of the GFP:Spri spots was in contact with the Rab5-positive vesicles representing the early endocytic compartment (**Fig 8B**). Starving animals for even a short time, led to an increased density of Rab5-positive vesicles, many overlapping with the inner part of the GFP:Spri spots (**Fig 8B and 8B’**). This was not observed when using endocytic syntaxin labeling as a control (**S3B Fig**). This suggests that the increase of a Rab5-positive sub-population of vesicle is unique to the starved conditions.

To provide a direct insight into *vap*-associated activity in these cells, we then measured the density of endocytic Rab5-positive vesicles in sections below the surface of Spri spots. Loss of *vap* was associated with elevated formation of vesicular Rab5 that was quite evident in the fed state (**Fig 8C**). This is consistent with perturbations of PI(3)P levels and confirms the constituent impinge of Vap on Spri. No further elevation of Rab5 was associated to the starved *vap* cells (**Fig 8C’**).

To show that local production of Rab5-GTP indeed involved the activity of the GEF Spri, we analyzed *spri-*null fat cells and found that Rab5 was strongly decreased in the case of fed cells (**Fig 8C**). On the other hand, starvation promoted usual elevation of endocytic Rab5-vesicles in the *spri* mutant cells (**Fig 8C’**). While this confirms our conclusion that starvation-induced autophagy may not require *spri*, it also suggests that other Rab5 GEF in this compartments are at work in this context (i.e. during starvation).

In summary, measuring the direct emanations of *vap* and *spri* activities in such an in-vivo setting argues that the two genes are primarily active in the fed state where they presumably contribute to basal autophagy.

### VII. Rab5 controls PI(3)P and phagophore formation primarily in fed cells

**Fig 9:**
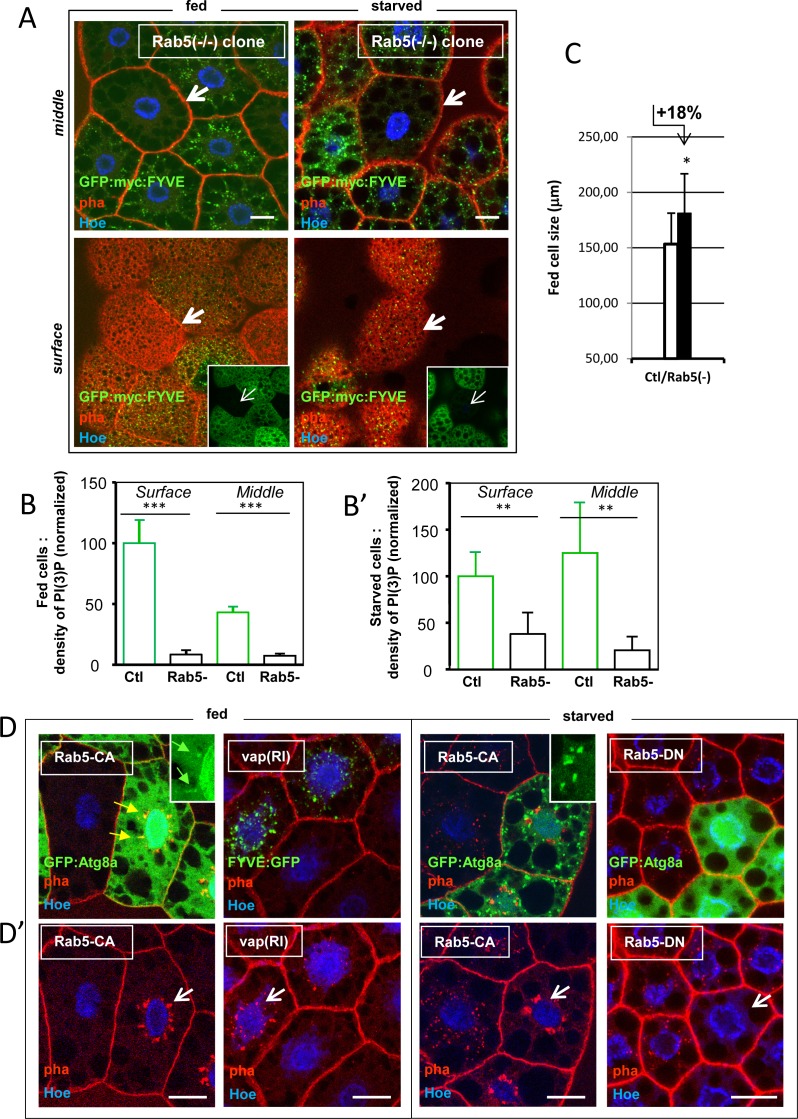
Rab5 is required for early events during autophagosome biogenesis. (A) Clones of fat body cell deprived of any Rab5 product were generated by heat shock-Flp/FRT mitotic recombination of the *Rab5*^*2*^-null, deletion allele, in fed or 2h-starved animals in presence of myc-tagged PI(3)P biosensor (GFP:myc:FYVE) expressed in every fat-body cells. Anti-myc immunostaining (in green) was used to detect the biosensor in fixed tissue. Inset: identified GFP-, *Rab5*-/- clones. In fed cells, both perinuclear and dispersed cytoplasmic PI(3)P is absent from mutant cells (arrows pointing two plans of same clones). Little but detectable PI(3)P labeling persists in the Rab5-/- cells of 2h-starved animals. Scale bars = 20 μm. **S8 Fig** for single channels. (B, B’) The *myc* labeling of tagged GFP:FYVE of surface or middle cell plans of *Rab5*-/- clones was quantified relative to neighboring control cells in both fed and starved cells samples of A (fed GFP- n = 3, fed GFP+ n = 6; sta GFP- n = 3, sta GFP+ n = 6). Fed, *Rab5*-null cells has negligible amount of PI(3)P. 2h-starved cell shows 1/3 to 1/8 of residual PI(3)P labeling in the *Rab5* mutant cells (surface or middle plans respectively). Error bars are standard errors; significances are from Student’s *t*-tests. (C) The cell size of *Rab5*-null cells is increased in identified clone of fed animals compared to neighboring wild-type cells. Data from A were quantified as in **Fig 3E** (Ctl n = 23; *Rab5*-/- n = 10). Error bars are mean differences; significance is from Student’s *t*-tests. (D-D’) Clones of *Rab5-CA*, *vap(RI)* or *Rab5-DN* cells were generated together with the *GFP*:*Atg8a* or GFP:FYVE markers expression, using the *Act>CD2>Gal4* flipout cassette method. Fed or 3h-starved fat cells of early/mid-3^rd^ instar were analyzed in fixed tissues. Fluorescent GFP and phalloïdin are shown in panel D, and phalloïdin-labeled actin structures are shown panel D’. In fed animals, *Rab5-CA* clones induces excessive actin-labeled phagophore network (or PhAS, white arrow in D’). These overlapped with GFP:Atg8a-labeled isolation membranes (yellow arrows in D or green arrows in inset). In the same conditions, *vap(RI)* clones leads to equivalent excess of actin-labeled PhAS formation (arrow). Upon starvation, *Rab5-CA* clones formed plenty of induced-autophagosomes. Those were abnormal in shape (inset: enlarged picture) and were not overlapping with the large PhAS network (arrow in D’). Analysis of the Rab5-CA-induced green-vesicle signal (Materials and Methods) revealed that mutant cells has an 2.8 time higher density in autophagosomes compared to wild-type controls; median size: *Rab5-CA*, Mdn = 0,931 μm^2^ (n = 411); control, *w-* Mdn = 1,238 μm^2^ (n = 158). Total amount of autophagy-membranes areas are thus doubling in the *Rab5-CA* expressing cells. Inhibition of Rab5 in *Rab5-DN* expressing clones prevents the formation of starvation-induced autophagosomes and that of endogenous actin-labeled PhAS (arrow in D’). Scale bars in all panels = 20 μm. Genotypes. (A) Assay: *w*^*1118*^*/ hsFLP*^*12*^*; Rab5*^*2*^, *FRT40A/ 2xUAS-EGFP*, *FRT40A*, *Fb-GAL4; UAS-GFP*:*myc*:*2xFYVE/+*. *(D)* Assay: *w*^*1118*^*/ hsFLP*^*12*^*; UAS-GFP*:*myc*:*Atg8a*, *Act>CD2>GAL4/ UAS-Rab5*^*CA*^. *w*^*1118*^*/ hsFLP*^*12*^*; UAS-vap(RI-KK)/ +; Act>CD2>GAL4*, *UAS-GFP*:*myc*:*2xFYVE/+*. *w*^*1118*^*/ hsFLP*^*12*^*; UAS-GFP*:*Atg8a/*, *Act>CD2>GAL4/ UAS-Rab5*^*DN*^.

The above results suggest a model whereby Rab5 is an important element in a chain determining basal autophagy rate. We thus assessed the contribution of *Rab5* to normal PI(3)P and autophagosome formation in this cell type. Clones of *Rab5*-null mutant cells generated in well fed animals, showed negligible PI(3)P compared to juxtaposed wild-type cells, as quantified in two different cell heights (**Fig 9A–9B’**). The same determination carried in starved animals showed that a fraction of PI(3)P would nonetheless form in the *Rab5*-null cells. However, no or rare autophagosomes were detected in these cells (**S6D Fig**). Identical conclusions were drawn from studies using a Rab5-dominant negative (DN) construct **(S6A–S6C Fig)**. Significant, alike clonal *spri-*inhibited cells, loss of *Rab5* conferred growth advantage to the mutant cells in fed conditions (**Fig 9C**). Hence, the gain of intrinsic size of mutant fat cells under fed conditions (or on contrary the cell size reduction), appears a distinctive feature of regulators involved in the generation of basal autophagy.

To determine if overall Rab5 activity would be limiting for the implementation of autophagy, we expressed activated Rab5 constructs (*Rab5-CA*) in clones of fed fat cells in the presence of the autophagosome membrane marker *GFP*:*Atg8a*. In this context *Rab5-CA* did not induced large autophagosomes over the cytosol (**Fig 9D**). Only presumed isolation membranes (autophagosome membrane precursors -or phagophores) were discernible at perinuclear locations (**Fig 9D** inset). These were overlapping large filamentous actin structures (labeled by phalloïdin) that were newly induced in the *Rab5-CA* expressing cells. Interestingly, silencing of *vap* in fed animal cell clones produced analogous induction of actin structures (**Fig 9D’**). Upon starvation, however, *Rab5-CA* cells formed abundant thought misshaped, autophagosomes membranes (**Fig 9D**). These data are consistent with the idea that activated Rab5 mimicked at least some of the *vap* mutant effects.

Interestingly, the generation of a phalloïdin-labeled network of branched actin combined to labeled isolation membranes is a signature of developing phagophore assembly sites (hereafter referred as PhAS) that were initially described at the omegasomes sites of starving cultured cells [67]. We thus verified the state of those PhAS in the fat body tissue. Consistent with the generation of microscopic autophagosome organelles within normal fed cells, we found that labeled pericentral actin was present in them in the form of discrete punctate. However, these structures were more visible upon starvation (**S6E’ and S6E” Fig** and [67]). Interestingly, we also found that *Rab5* activity was required for pericentral actin formation in both fed and starved cells (**S6B” Fig and Fig 9D’**). We conclude that *Rab5* is initially important for the formation of PhAS in fed cells and that these structures remained absent in the mutant cells experiencing starvation. Conversely, the excess of actin-based PhAS structures, as induced by activated *Rab5* in fed cells persisted when these were subjected to starvation (**Fig 9D’**).

In summary, Rab5 activity is primordial to phagophore formation in fed cells, but its requirement likely extends to starving cells where these structures became enlarged (note that, a strict requirement for *Rab5* only during the course of starvation, could formally not be assessed using the current experimental setting, **Fig 9B”**). In line with these conclusions, *Rab5* was required to generate any PI(3)P in fed cells (**Fig 9A**), agreeing with previous demonstration showing that formation of PI(3)P *in-vivo* is essential for actin punctate generation, while *in-vitro* actin polymerization was stimulated by PI(3)P in a concentration-dependent manner [67].

## Discussion

While centered on fat tissue, our study of the role of *vap* supports the general conclusion that a heightened rate of basal autophagy can explain all currently known *vap* mutant phenotypes. This is the first report of a mutant targeting basal autophagy as such, thought the nuclear protein Acinus had been proposed to impinge on basal autophagy levels in a TOR-independent fashion [68]. Because of a lack of convenient markers to assess the state of basal autophagy directly, our investigations relied on its deregulations as displayed by mutants (but see **S7 Fig** for comments). The normal status of autophagy in 3^rd^ larval instar fat bodies was disturbed, showing ectopic incidence of lysosome-like structures in fed animals. This correlated with a reinforcement of the starvation-induced autophagy program. In the same line, we documented that PI(3)P phosphoinositide was constitutively upregulated in *vap* fat cells, particularly when we considered the pool of PI(3)P being at the origin of autophagosome membranes.

We showed that *vap* fat cell clones and whole mutant animals grew with intrinsically reduced fitness characteristics, leading to mild reductions of cell and organism sizes. Increased cellular expanses and disfavored competitive cell growth are hallmarks of autophagy-active cells [14, 16]. The net growth-suppressive effect of autophagy has been discussed both in terms of the energetical impact of synthesizing the degradative machinery, and its reverse coupling to biosynthetic processes such as mTOR-driven protein synthesis [16]. Interestingly, undergrowth of *vap* cells was independent of *Atg1* activation, suggesting that the establishment of basal and starvation-induced autophagy relies on distinct mechanisms [69]. Consistent with this, TOR-signaling remained unchanged in the mutant context. One possibility is that normal turnover of active Vps34-core complexes underlies the physiological rate of basal autophagy.

We established that *vap*-dependent starvation-hypersentivity defect was in large part accounted by the developmentally-sustained elevation of basal autophagy rate, which would drive a wasteful energy balance and a low nutritional status of the adult flies. Indeed, phenotypic rescue of this defect by wild-type Vap expression likely counterbalanced the elevated basal autophagy rate, returning to a more natural level. Because the excess of Vap was by no way constrained in this experiment, inhibition of the standard basal autophagy level could be ensuing, resulting in extra resistance to starvation (see **S4H Fig**). Indeed, Vap over-expressing cells showed favored cell-growth characteristics. Likewise, *spri-* cells had an increased cell-size phenotype and mutant *spri* also were associated with extra starvation resistance (**Fig 7**). Thus, basal autophagy could be genetically manipulated to an elevation or to a reduction, resulting in cell undergrowth or slight overgrowth effects, and to corresponding changes of organism’s health and fitness. We believe that deregulated basal autophagy could disturb other tissues, impairing metabolism and organ efficacy. For instance, we note that *vap* larvae and adults had diminished locomotor activities. Surely enough, a sustained elevation of basal autophagy in neurons is a likely cause of the age-dependent brain neurodegenerative phenotype found in the fed mutant adults [46]. Indeed, dying neurons were filled with ectopic autolysosomes and had morphological characteristics of type II cell death [46]. These considerations may therefore set limits to the use of strategies based on autophagy stimulation as a cure for neurodegenerative disorders characterized by accumulation of aggregation-prone protein [7]. More studies of the consequences of modulating the rate of basal autophagy in given tissues and organs would help uncover other possible detrimental effects.

*Vap* was additionally required for normal progression of starvation-induced autophagy. This was not expected given the finding that *vap* and *spri* are primarily active in fed cells. However, it could be explained if the formation of new, starvation-induced autophagy membranes, was coupled to basal autophagy in the fed cells. Significant to this, we saw overtly expanded autophagosomes and lysosomes membranes in result of excessive PI(3)P-dependent membrane influx in the starving *vap* cells (**Fig 4A and 4A’**). Note that, when analyzed on a short starvation period, this phenotype likely results from a fast ‘on rate’ of membranes formation rather than a so-called ‘off rate’, referring to membrane accumulation. Thus, autophagy membrane biogenesis *per se* could be the focus of PI(3)P deregulation. Excessive membrane influx in turn led to aberrant vesicular fusions and the blocking of autophagosome-lysosome fusions. The latter phenotype is analogous to the default autophagosome-vacuole fusions in *Saccharomyces cerevisiae* mutants lacking specific PI(3)P phosphatases [65, 70].

In support of the coupling hypothesis, we found that the heightened levels of autophagy-related PI(3)P in fed *vap* cells remained after starvation (**Fig 2B**). On the contrary, the density of endocytic Rab5-vesicles did not exceed that of controls in the starved *vap* cells (**Fig 8C’**). Taken together, these observations suggest that the elevated PI(3)P generated under fed conditions (or remaining structures containing it), perdures, thus confounding the newly induced autophagy-membrane dynamics. A natural coupling process may underlie the transition of wild-type cells from a basal to an induced state of autophagy.

Sprint behaved as the catalytic sub-unit of Vap/Spri complexes. We infer that the endocytic Rab5-GEF activity of Spri is essential to the generation of *vap*-dependent hypersensitivity to starvation (**Fig 7**), as unbound Spri moieties (i.e. the situation in *vap* mutants) caused unusual accumulation of endocytic Rab5 vesicles (**Fig 8C**). In turn, this likely is at the origin of excessive Vps34 recruitment and PI(3)P production. Thus, Vap-restrained Spri activity controls proper basal autophagy rate in fed animals through an endocytic, Rab5-positive, effector route. Consistent, *spri* has also been proposed as a pertinent mediator of the neuroprotective effect of *vap* [48]. A requirement for *spri* during starvation-induced autophagy could not be excluded. *Spri* flies were not sensitive to starvation, though potential redundancies with other Rab5 GEF(s) of the endocytic compartment may hide such a requirement [71]. In these conditions, we nonetheless saw receded autophagy vesicles and abortive fusion events happening during the mutant autophagy process (**Fig 6A–6D**). In contrast to our observations in *vap* cells, we argue that the small-sized autophagosomes and lysosomes in *spri* cells arose from weakened endomembrane influx. This is supported by the extensive reduction of vesicular Rab5 production and PI(3)P production found in the *spri* fed cells (**Fig 8C and Fig 6E**).

Still other members of the Vps9-domain Rab5 GEF(s) were found effective in endocytosis and for the generation of fusion-competent vesicles, leading to proper implementation of (at least) endosomal trafficking [71]. A previous study using *Caenorhabditis elegans* found a level of redundancy between two different Vps9-domain GEF proteins involved in receptor-mediated and fluid phase endocytosis [72]. During this work, we noticed singular parallels between the altered autophagy membrane dynamics in *vap* mutants (i.e. resulting from excess Spri), and that resulting from GOF expression of wild type or pathogenic forms the GEF called Alsin [73]. Because mutated Alsin proteins were responsible of the juvenile form of familial ALS2 disease (amyotrophic lateral sclerosis 2) [74], it should be interesting to ask if *Drosophila ALS2* can influence vesicular Rab5 production or basal autophagy rate in neuronal cells. This can easily be tested using the fat body system as read out.

### Concluding remarks

**Fig 10:**
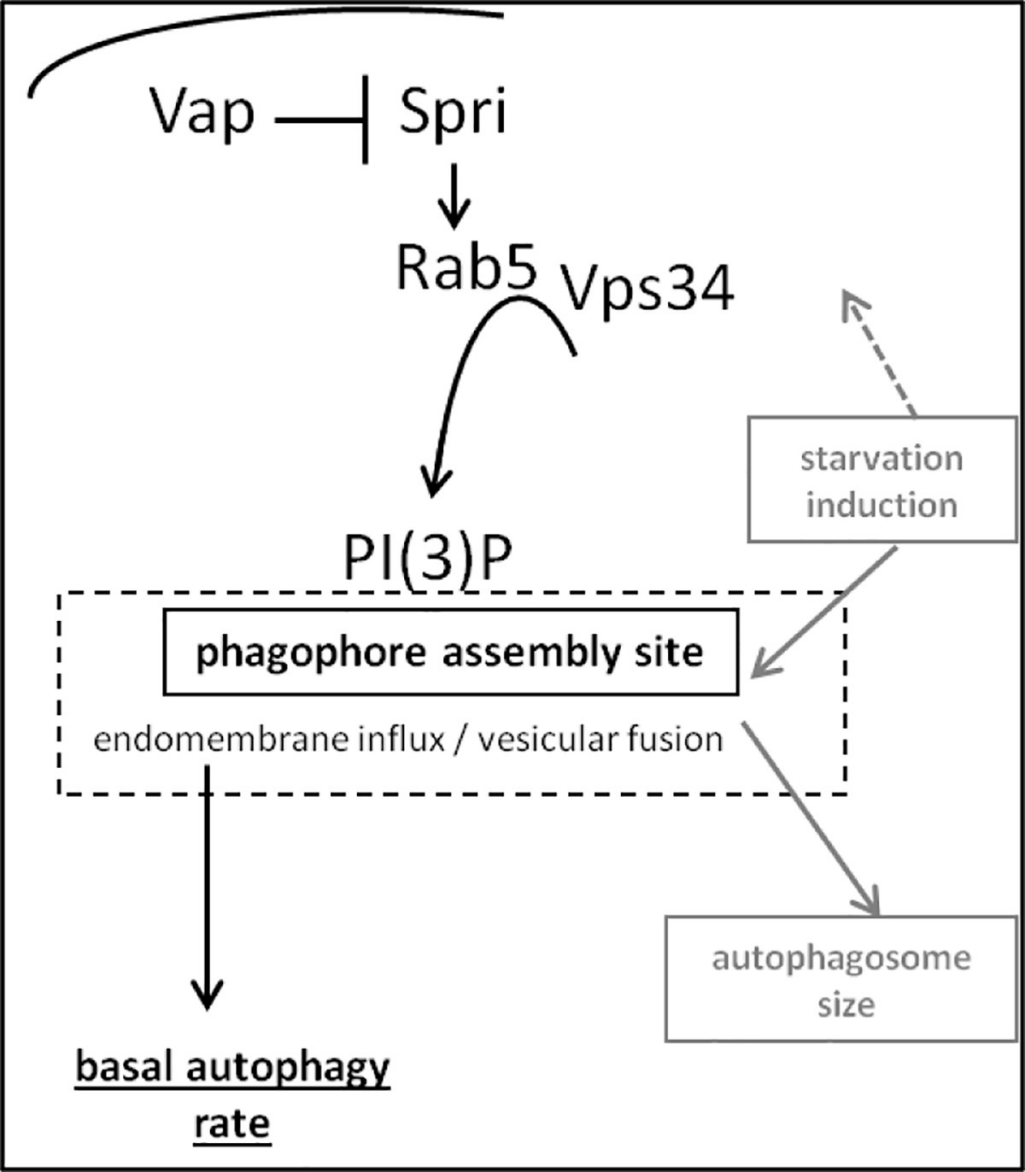
A model for the generation of basal autophagy and its coupling to stimulated autophagy. In fat body tissue, endocytic cell-compartment contains Rab5-positive vesicles issued partly by the activity of Vap/Spri modules. Here, the RasGAP homolog Vap (*Vacuolar Peduncle)*, negatively regulates the Rab5-GEF partner, Spri *(Sprint)*. Autophagy competent Rab5-vesicles drive nucleation of pre-autophagosome structures evolving into phagophores at multiple ER sites called omegasomes. This sets the foundation of the double-layered membranes of the autophagosome organelles. Rab5-vesicles “competence” presumably involves the recruitment or activation of proautophagy-competent Vps34 complexes (or Vps34-complex I) and translocation to omegasomes, ending in a local stimulation of PI(3)P synthesis and nucleation of proportionate PAS and phagophore components [76] (i.e. higher PI(3)P synthesis promotes larger phagophores; see text). In normal fed cells, microscopic autophagosomes are manufactured from extant phagophores and these assume a basal autophagy rate (black arrow downward). On starvation, Rab5-vesicle density is increased (dashed grey arrow) causing further phagophore inflation, which is modeled on its extant architecture. Starvation-induced autophagosome size is therefore a reflection of fed-cell phagophore size. The parallel formation of endosomal membranes is not represented.

Here, we provide evidence for a novel trafficking connection between an endocytic regulator showing GAP homology (Vap) (see **S5 Fig**) and a bound catalytic GEF (Sprint), which in the inner cell surface is generating a flow of small Rab5-positive vesicles (also called primary endocytic vesicles). In addition to founding endosomes following homotypic fusions [33], we suggest that these vesicles are contributing an essential role in the establishment of basal autophagy rate, presumably by promoting the formation of actin and isolation membrane arrays of the PhAS. While we propose that the architecture of those PhAS is a relevant focus of the *vap*-induced autophagy deregulation, we note that a general PI(3)P deregulation may as well impact later stage of vesicular biogenesis, such as membrane elongation, autophagosome completion, or endosomal vesicle homeostasis. We do observed ectopic fusions resulting from aberrantly fused membrane compartments in parallel to expanding lysosomes compartments (**Fig 4A–4D”**). Also, we cannot exclude that disruption of endosomal PI(3)P may play a role in the observed basal autophagy defect, though we think it less likely. Finally, as noted before [75], we propose that implementation of basal autophagy is coupled to the biogenesis of the large starvation-induced autophagosomes (**Fig 10**).

Our evidence are indicating that *Rab5* activity is required for PI(3)P formation and the determination of ER-associated phagophores at early stages of autophagosome biogenesis (**Fig 9A, 9D and 9D’**). Based on phenotypic similarities, active Rab5 serves as a likely downstream effector of *vap* (**Fig 9D and 9D’**). However, these data by no means, are showing how this is achieved. It is possible that Rab5 vesicles issued from Vap/Spri complexes are involved in the launching, assembly or activation of autophagy-competent Vps34 complexes (the Vps34-complex I). It was already suggested that Rab5-positive endosomal vesicles could be directly delivering the necessary machinery for initiation of autophagy at omegasome sites [36]. This would thereafter lead to the localized generation and accumulation of PI(3)P and subsequent nucleation of early PAS components [76]. Indeed *vap* activity functions as a clear antagonist of Vps34 proautophagic activity (**S2A Fig**). That *Rab5* is taking part of early autophagosome biogenesis is also consistent with previous analysis that used cell culture model of Huntington disease to study the relevance of Rab5-Vps34-Beclin1 complexes in the autophagy-mediated clearance of toxic huntingtin [38]. Consistent with our results, inhibition of Rab5 in this system caused abnormal progression into the phagophore stage, which immediately follows PAS formation [38].

While our investigations described simple properties of basal autophagy, we found an unexpected link between autophagosome biogenesis ongoing in fed and in starved cells. As discussed above, the persistence of PI(3)P or phagophore-related material, could well be supporting such a link. Interestingly, recent advance on early steps of autophagosome formation found that PI(3)P-regulated actin bundles were forming inside the isolation membranes and acted as a driving force for autophagosome membrane expansion, conditioning their final shape and size [67, 77]. Here, we found that phagophore formation is directed by Rab5 activity and may actually be proportional to it (consider for instance the perinuclear actin spots in the fed, starved or *Rab5-CA* cells, **S6E’ Fig and Fig 9D’**, and gross endocytic Rab5 activity in the same situations, **Fig 8C and 8C’ and Fig 9D’**). We thus speculate that final autophagosome size is dependent on initial fed-cell phagophore architecture, the latter being a reflection of the endocytic Rab5 vesicle density (**Fig 10**).

A previous report described an analogous mechanism of autophagosome sizing in cultured cells. Here, maturation of fed-cell early autophagosome precursors involved expanding endocytic Atg16L-positive vesicles undergoing homotypic fusions into normally-sized phagophores. Agreeing with our observation, experimental modulations of fed-cell precursor (and phagophore) size, impinged on the size of the starvation-induced autophagosomes [75]. Hence, proper architecture of the initial phagophore not only supports essential function in the well fed cells (i.e. for regulated basal autophagy rate), but also sets a prepattern used for the response to autophagy-inducing stimuli.

## Supporting information

S1 Fig Quantification of acidic compartments, PI(3)P, p62 and Rab7:GFP markers in *vap* mutants. (A) The size distribution of LysoTracker-positive punctate of **Fig 1D** was plotted as particle number in every 0.1 μm^2^ size increments. Mutant, *vap*^*1*^ fat bodies has greater number of stained particles, which are spanning larger size range when compared to control *w-*.
(B) Quantification of absolute GFP:FYVE signal of perinuclear and cytoplasmic areas of **Fig 2A**. *vap* cells has significant elevations of the dispersed cytoplasmic PI(3)P pool (p<0.001) and perinuclear PI(3)P pool (p<0.05), both in the fed and starved conditions as compared respective controls. When subjected to starvation, control and mutant cells show a 3–4 time elevation of the GFP:FYVE signal (p<0,0001), indicating that starvation *per se* resulted in expanding PI(3)P-membrane influx. Error bars are mean differences; significances are from Student’s *t*-tests.
(C) Determination of total PI(3)P phosphoinositide by Competitive ELISA using whole lipid extracts of fed and 3h-starved mid-3^rd^ larvae (Materials and Methods). Absolute estimates in the mutant, *vap*^*1*^ animals are greater than those found in three reference strains used as controls: *carnation (car*^*1)*^, *Oregon-R (Or)* and *white (w-)* (n = 3 except for car, n = 2). Each assay were the equivalent to 2.5 larvae. Error bars are standard deviations; significances are from Student’s *t*-tests.
Genotypes. (C) Control: *car*^*1*^*/Y*. *+/Y (Oregon-R)*. *w*^*1118*^*/Y*. Assay: *vap*^*1*^*/Y*.
(D,D’) The size distribution of starvation-induced p62 bodies and Rab7:GFP aggregates of **Fig 4B** were analyzed in boxplots. p62 bodies and Rab7:GFP aggregates of mutant, *vap*^*1*^ cells are of a larger size range compared to control, *w-* cells. p62 bodies: (*w-* n = 357, Mdn = 0,27 μm^2^; *vap*^*1*^ n = 741, Mdn = 0.43 μm^2^). Rab7:GFP (*w-* n = 122, Mdn = 0.57 μm^2^; *vap*^*1*^ n = 367, Mdn = 0.67 μm^2^). Medians are drawn as thick lines; significances are from Mann Whitney test.
(TIF)

S2 Fig Antagonism between *vap*-dependent PI(3)P regulation and Vps34 activity, and TR-avidin accessible compartment in *vap*. (A) Clonal overexpression of wild-type *UAS-dVps34* transgene using the *Act>CD2>Gal4* flipout cassette method, causes wider dispersion of PI(3)P in fed and 3h-starved cells compared to control, *w-*, as revealed by co-expressed GFP:FYVE biosensor probe. These effects are phenocopies of the loss of *vap* in fed and starved fat cells respectively (see **Fig 2A**). Scale bar = 20μm.
(B) Inhibition of Vps34 using *cg-Gal4*-driven expression of dominant negative Vps34 (*UAS-dVps34*^*DN*^) in fat cells abolishes co-expressed GFP:FYVE staining in control, *w-* and *vap*^*1*^ contexts, both in fed and in1h30’-starved cells. Scale bars = 20μm.
(C) Quantification of perinuclear versus cytoplasmic areas of stained FYVE probe was performed following a setup described in Juhàsz *et al*. [34] and illustrated here. Shown are *GFP*:*Atg8a* and myc(only)-tagged FYVE expressed in fed or starved fat cells, after immunostaining detection of myc (in red). In fed cells, the manually delimited red ring (2–4 μm around nuclei) comprised the perinuclear early endosomes. In starved cells, the delimiting red ring isolated inner endosomes from outer green and red labeled autophagosomes forming in the cytosol. When autophagosomes are not labeled, this method practically distinguished the two FYVE probe-labeled populations with about 90% accuracy. Images on the right were manipulated to enhance the stained structures. Scale bar = 10μm.
(D) Clones of control, *w-* or *vap* RNAi-depleted cells, *vap(RI)*, were generated in the presence of the GFP:FYVE probe expression, using the *Act>CD2>Gal4* flipout cassette method and tissue subjected to *ex-vivo* TR-avidin incorporation (Materials and Methods). *vap(RI)* cells (marked by the GFP:FYVE) has increased labeled TR-avidin accessible compartment or perinuclear early endosomes (white arrows). Arrows in yellow point to the near complete overlap of the labeled tracer (red) and GFP:FYVE-labeled early endosomes (green) in control and mutant cells. Scale bar = 20 μm.
Genotypes. (A) Control: *w*^*1118*^*/ hsFLP*^*12*^*; UAS-GFP*:*myc*:*2xFYVE*, *Act>CD2>GAL4/+*. Assay: *w*^*1118*^*/ hsFLP*^*12*^*; UAS-GFP*:*myc*:*2xFYVE*, *Act>CD2>GAL4 /UAS-Vps34*^*wt*^. (B) Control: *w*^*1118*^*/Y; cg-GAL4/ UAS-Vps34*^*DN*^*; UAS-GFP*:*myc*:*2XFYVE/ +*. Assay: *vap*^*1*^*/Y; cg-GAL4/ UAS-Vps34*^*DN*^*/+; UAS-GFP*:*myc*:*2XFYVE/ +*. (C) *w*^*1118*^*/ hsFLP12; UAS-myc2*:*XFYVE/ +; Act>CD2>GAL4*, *UAS-GFP*:*Atg8a /+*. *(D)* Control: *w*^*1118*^*/ hsFLP*^*12*^*; UAS-GFP*:*myc*:*2xFYVE*, *Act>CD2>GAL4/+*. Assay: *w*^*1118*^*/ hsFLP*^*12*^*; UAS-vap(RI-KK)/ +; UAS-GFP*:*myc*:*2xFYVE*, *Act>CD2>GAL4/+*.
(TIF)

S3 Fig Clonal fat cell growth under starvation, density of endocytic control vesicles and TOR- signaling in *vap* mutant fat bodies. (A) Compared to clonal growth in fed conditions (**Fig 3A and 3E**), the relative size reduction of clonal *vap*^*1*^ fat cells versus control is not markedly different when animals grew under chronic starvation for ca. 88h (i.e. aa-poor food, Materials and Methods). Clones of *Atg1(RI)* mutant fat cells were analyzed in animal grown under the same chronic starvation for ca.88h. *Atg1(RI)* cells in this case, shows competitive growth advantage compared to control neighboring cells, as expected from autophagy-defective cells under starvation [14]. This data verified our chronic starvation conditions and the *Atg1(RI)* lines used in **Fig 3**. (Ctl n = 16, *vap*^*1*^ n = 8; Ctl n = 14, *Atg1(RI)* n = 13). Genotypes were as in **Fig 3.** Error bars are mean differences; significances are from Student’s *t*-tests.
(B) Average populations of endocytic-compartment vesicles were analyzed after labeling for syntaxin 7/12 / *D*.*melanogaster Avalanche* (*Avl*). Densities of endocytic Avl vesicles (number of vesicle per μm^2^ of cell area) were quantified form plan surface views of control *w-* and *vap*^*1*^ fat cells, in fed and starved conditions as in **Fig 8B–8C’**. Avl-positive vesicle densities remains relatively even after starvation in control, *w-* or *vap* mutant conditions (*wt (w-)* fed n = 2; *wt (w-)* sta n = 2; *vap*^*1*^ fed n = 4; *vap*^*1*^ sta n = 3). Error bars are standard errors; significances are from ANOVA.
(C) The activation of the *unk-LacZ* reporter construct was used to search for any devaluation of TOR-signaling in fat bodies of fed mutant *vap* animals. Images are immunostaining detection of LacZ expression. No staining of the reporter is observed in fed *vap*^*1*^*/Y* males larvae. On the other hand, reporter activation is readily obtained in tissue of 4h-starved mutant animals, attesting for normal inhibition of TOR-signaling and thus activation of the stress response factor REPTOR, which in turn mediates *unk* transcription [56]. Both negative and positive controls were obtained using fat bodies of hetererozygous, *vap*^*1*^/+ females larvae, where the *unk-LacZ* reporter is fully silenced in fed animals or fully induced in 4h-starved animals. Scale bar = 100 μm.
Genotypes. (A) Assay: males *vap*^*1*^*/Y; unk-LacZ /+*. Control: *female vap*^*1*^*/w*^*1118*^*; unk-LacZ /+*.
(TIF)

S4 Fig Vap-dependent starvation sensitivity and phenotypic rescue of *vap* function. (A) Aged-matched, 3-days old mutant, *vap*^*1*^ and *vap*^*2*^ males exhibited robust hypersensitivity to acute starvation (white arrow), as 50% of them are not surviving for longer than 36h (see Materials and Methods for assay). Control, *w-* and Oregon-R, Or strains resist for a longer period. Female genotypes showed the same effects.
(B) 13 days-old *vap* mutant flies shows an hypersensitivity-to-starvation phenotype similar to 3-days old flies despite the fact that mutant state has induced brain neurodegeneration for already 6 days in these flies [46].
(C) Flies fed with 15% sucrose only, are relieved from sensitivity to starvation whether mutants or controls.
(D) Two *D*.*melanogaster* RasGAP family members distinct of Vap are ineffective at rescuing starvation sensitivity of *vap*^*1*^ mutants (D, white arrows) when expressed using the ubiquitous *arm-Gal4* driver and corresponding transgenic wild-type constructs, *UAS-NF1* and *UAS-GAP1* [44].
(E-H) Partial rescue of starvation sensitivity is obtained after the restitution of *vap* activity using an *UAS-vap(wt)* transgene, and the fat body/hemocyte driver *cg-Gal4* (*E*, white arrow). The same relative rescue is observed using the fat body/midgut driver *ppl-Gal4* (not shown). Wider expression of *vap* using the *arm-Gal4* driver, effect an almost complete rescue (F, white arrow). The same result is obtained using the ubiquitous driver *Act-Gal4* (not shown). A complete rescue is associated with pan-muscular expression of *vap* using the *Mhc-Gal4* driver (G, white arrow) whereas pan-neuronal expression of *vap* as driven by *elav-Gal4*, results in a rescue exceeding the limit of the *w-* controls suggesting enhanced resistance to starvation (H, white arrow).
Genotypes. (A, B, C) Control: *+/Y (Oregon-R)*. *w*^*1118*^*/Y*. Assay: *vap*^*1*^*/Y*. *vap*^*2*^*/Y*. (D-H) Included *w*^*1118*^*/Y*. *vap*^*1*^*/Y*. as control reference (D) Control: *vap*^*1*^*/Y; arm-GAL4/+*. Assay: *vap*^*1*^*/Y; arm-GAL4/ UAS-NF1/+*. *vap*^*1*^ */Y; arm-GAL4/ UAS-GAP1/+*. (E) Control: *vap*^*1*^*/Y; ppl-GAL4/+*. Assay: *vap*^*1*^*/Y; ppl-GAL4/ UAS- Vap*:*myc*^*16*.*4*^*/+*. (F) Control: *vap*^*1*^*/Y; arm-GAL4/+*. Assay: *vap*^*1*^*/Y; arm-GAL4/ UAS-Vap*:*myc*^*16*.*4*^*/+*. (G) Control: *vap*^*1*^*/Y; Mhc-GAL4/+*. Assay: *vap*^*1*^*/Y; Mhc-GAL4/ UAS-Vap*:*myc*^*16*.*4*^*/+*. (H) Control: *vap*^*1*^*/Y; elav-GAL4/+*. Assay: *vap*^*1*^*/Y; elav-GAL4/ UAS-Vap*:*myc*^*16*.*4*^*/+*.
(TIF)

S5 Fig Vap-domains analysis, quantification of PI(3)P in *spri* mutants and semi-quantitative TEM analysis in *vap* mutants. (A) A structure-function analysis of Vap domain mutants was performed based on the capacity of over-expressed Vap to antagonize fat body-cell PI(3)P as shown in **Fig 2B**. For each expressed constructed, a visual estimates of PI(3)P-inhibition was determined using 3 images of 3–4 cells wide clones. Maximal inhibitory activity of wild-type Vap (wt) is denoted as (+++), while GAP catalytic mutant GAP*, and the SH2*32*, SH2*32 and SH23*2 mutants are inactive at eliminating PI(3)P vesicles, and thus denoted as (-). The N-terminal fragment SH232, comprising the two SH2 domains is almost as active as wild type, which is denoted as (++). Activity of Vap (wt) and of the SH232 construct also inhibited LysoTracker staining (**Fig 2C**). Together, these analysis shows that SH2-domains are necessary and sufficient to suppress PI(3)P formation in fat body cells. Note that integrity of the GAP catalytic domain is essential for PI(3)P-inhibitory activity only when mutated as part of the full length protein. This is in accordance with the “Ras-effector model” of signaling proposed for human p120RasGAP activation [1*, 2*].
(B) Quantification of GFP:FYVE signals of perinuclear and cytoplasmic PI(3)P pools from **Fig 6E** (performed as in **Fig 2**). Mean GFP area signals (left chart) or mean foci number (right chart) of selected cells were calculate for *w-* control clones (fed n = 8 plans of 4 cells; sta n = 10 plans of 5 cells) and *spri(RI)* clones (fed n = 12 plans of 6 cells; sta n = 10 plans of 5 cells). The difference between the two representations is accounted by the small size of the *spri-* foci. Error bars are standard errors; Significances are from Student’s *t*-tests.
(C) Characteristic vesicular autophagy-structures were compared in equivalent number of TEM sections of control, *w-* and *vap*^*1*^ starved fat tissues (Materials and Methods and **Fig 4D**). Note the accumulation of autophagosomes and amphisomes structures in the *vap*^*1*^ mutant, consistent with a block of the autophagy flux at late maturation stage (see text).
Genotypes. (A) Control: *w*^*1118*^*/ hsFLP*^*12*^*; UAS-GFP*:*myc*:*2xFYVE / UAS-Vap*:*myc*^*16*.*4*^*; Act>CD2>GAL4/+*. Assay: *w*^*1118*^*/ hsFLP*^*12*^*; UAS-GFP*:*myc*:*2xFYVE/ +; Act>CD2>GAL4 / UAS-Vap*:*myc*^*R695K*^. *w*^*1118*^*/ hsFLP*^*12*^*; UAS-GFP*:*myc*:*2xFYVE / +; Act>CD2>GAL4; Vap*:*myc*^*22*.*2*^. *w*^*1118*^*/ hsFLP*^*12*^*; UAS-GFP*:*myc*:*2xFYVE / UAS-Vap*:*myc*^*N15*.*1*^*; Act>CD2>GAL4/+*. *w*^*1118*^*/ hsFLP*^*12*^*; UAS-GFP*:*myc*:*2xFYVE / UAS-Vap*:*myc*^*B59*.*1*^*; Act>CD2>GAL4/+*. *w*^*1118*^*/ hsFLP*^*12*^*; UAS-GFP*:*myc*:*2xFYVE/ +; Act>CD2>GAL4/ UAS-Vap*:*myc*^*17*.*3*^.
1*. Martin G a, Yatani a, Clark R, Conroy L, Polakis P, Brown a M, et al. GAP domains responsible for ras p21-dependent inhibition of muscarinic atrial K+ channel currents. Science 1992; 255(5041): 192–4.
2*. Tocque B, Delumeau I, Parker F, Maurier F, Multon MC, Schweighoffer F. Ras-GTPase activating protein (GAP): A putative effector for Ras. Cellular Signalling. 1997. p. 153–8.
(TIF)

S6 Fig Additional data on the requirement of *Rab5* for normal autophagosome biogenesis. (A-B”) Control, *w-* and dominant-negative *Rab5* expressing cell clones were generated using the *Act>CD2>Gal4* flipout cassette method together with the GFP:FYVE marker. Fed or 3h-starved fat cells of early mid-3^rd^ larvae were analyzed in fixed tissues focusing either in middle cell or below the cell surface close to cortical actin.
Control, *w-* clones shows normal PI(3) P distribution in fed or starved fat cells (A).
In fed cells, *Rab5-DN* prevents the formation of PI(3)P at pericentral and cell surface locations. Instead, fluorescent GFP:FYVE probe remains throughout the cytosol. In starved cells, residual PI(3)P vesicles are apparent in the cytosol and in the cell surface (B-B’). *Rab5-DN* clones are missing the pericentral actin network in fed and starved cells (arrows) as analyzed by phalloïdin staining (B”).
(C) *Rab5-DN* clones are associated with expression of *GFP*:*Atg8a* marker (image from **Fig 9D**). No GFP-labeled autophagosomes are detected in the starved *Rab5-DN* expressing cells and fluorescent probe remains over the cytosol (**Fig 4A and Fig 6A** for GFP-labeled autophagosomes).
(D) *Rab5*^*2*^-null fat-cell clones were generated by mitotic recombination as in **Fig 9A,** in fed and 3h-starved animals and analyzed for the presence of mCherry-labeled autophagosomes using p-*mChAtg8a* made of the natural *Atg8a* promoter (Materials and Methods) (insert: identified GFP-, Rab5-/- clones). Not any red-labeled autophagosomes are present in fed cells and rare mCherry-labeled structures are associated with the Rab5-/- clone in starved cells (arrows) compared to neighboring wild-type cells. Note the large-sized *Rab5* mutant cells in A through D.
(E-E”) Control, *w-* cell clones is associated with GFP:FYVE marker expression and analyzed for phalloïdin staining, revealing thin pericentral punctuate structures in all fed cells and larger ones in all 3h-starved cells (E’ inset: higher magnification images). Image stacks helps to visualized the actin structures in fed cells (E”). Scale bars in all panels = 20 μm.
Genotypes. (A-B’) Control: *w*^*1118*^*/ hsFLP*^*12*^*; UAS-GFP*:*myc*:*2xFYVE*, *Act>CD2>GAL4/+*. Assay: *w*^*1118*^*/ hsFLP*^*12*^*; UAS-GFP*:*myc*:*2xFYVE*, *Act>CD2>GAL4/ UAS-Rab5*^*DN*^. (C) *w*^*1118*^*/ hsFLP*^*12*^*; Act>CD2>GAL4*, *UAS-GFP*:*Atg8a/ UAS-Rab5*^*DN*^. (D) *w*^*1118*^*/ hsFLP*^*12*^*; Rab5*^*2*^, *FRT40A/ 2xUAS-EGFP*, *FRT40A*, *Fb-GAL4; p-mChAtg8a/+*. (E-E”) Control as in A.
(TIF)

S7 Fig Fed mutant *vap* cells display markers of autophagy resembling stimulated autophagy. The state of endogenous p62/SQSTM1 autophagy-flux marker and late endosome Rab7:GFP marker were recorded in well fed cells of control, *w-* and mutant, *vap*^*1*^ fat bodies as in **Fig 4B**. These were compared to starved control, *w-* cells (image from **Fig 4B**). Fed mutant and starved control cells shows elevated number of p62 bodies and non-overlapping aggregates of Rab7:GFP, suggesting that autophagy is at least partially stimulated in the fed *vap* mutant conditions. Scale bar = 10 μm.
(TIF)

S8 Fig Single channel views of Fig 9A. Scale bars = 20 μm.
(TIF)

S1 Table A numerical evaluation of *spri(RI)* phenotypes shown in Fig 6. Note that analyzed autophagy defects of *spri(RI)* fat cells were always showing incomplete penetrance (n = 30–40 analyzed cells). This feature is likely in relation to the variegating nature of the *spri* gene [52] or the RNAi construct.
(TIF)

S1 File Raw-image files corresponding to the fed panel of S8 Fig (ch1 = Hoechst, ch2 = GFP clonal marker, ch3 = anti-myc, ch4 = phalloïdin 647H conjugate). (ZIP)

S2 File Raw-image files corresponding to the starved panel of S8 Fig (ch1 = Hoechst, ch2 = GFP clonal marker, ch3 = anti-myc, ch4 = phalloïdin 647H conjugate). (ZIP)
