# Supplementary figures and images for: Control of basal autophagy rate by vacuolar peduncle
Source: PLoS One. 2019 Feb 8;14(2):e0209759. doi: 10.1371/journal.pone.0209759 (PMC6368412; doi:10.1371/journal.pone.0209759)

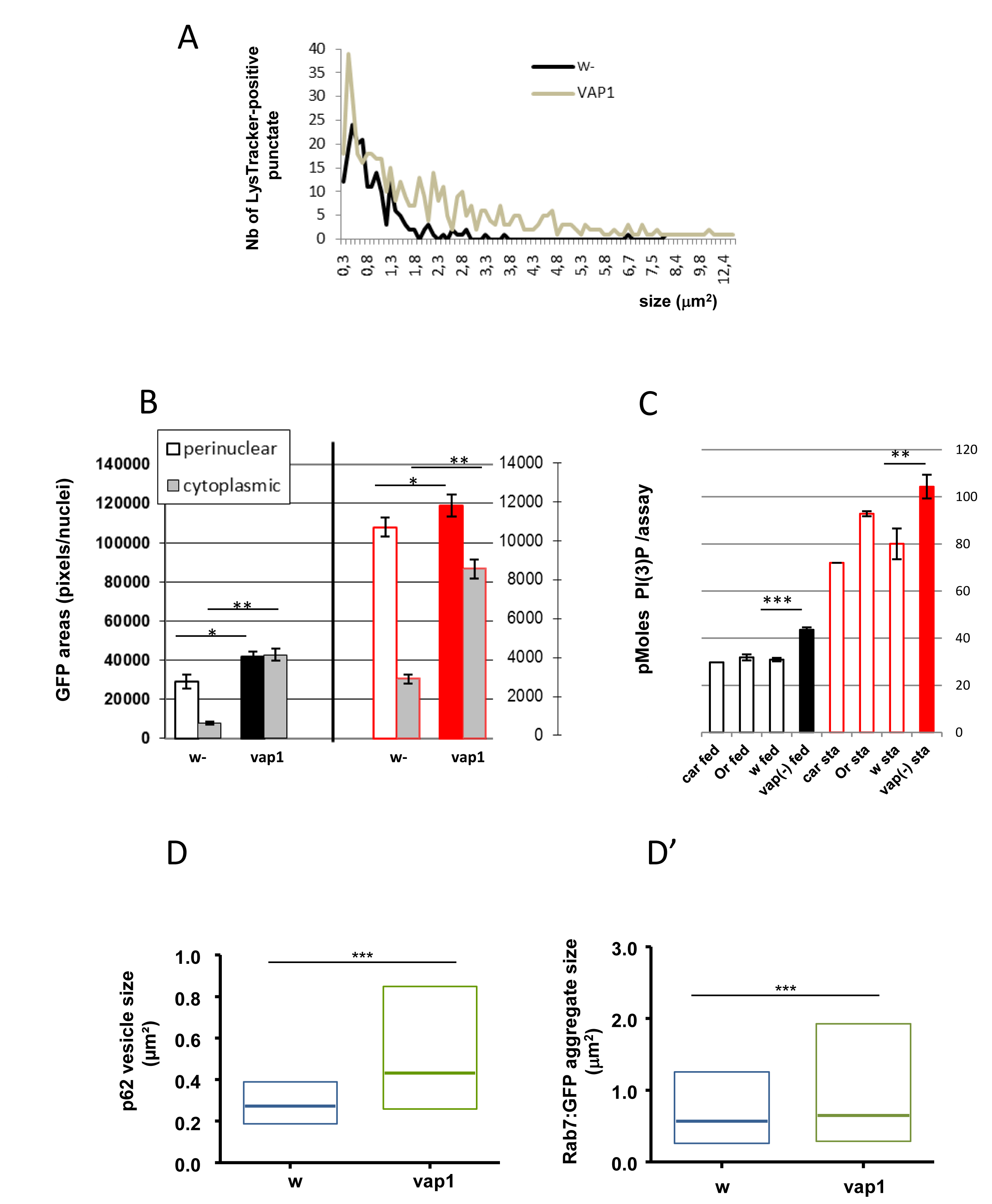

Supplement: S1 Fig — (A) The size distribution of LysoTracker-positive punctate of Fig 1D was plotted as particle number in every 0.1 μm2 size increments. Mutant, vap1 fat bodies has greater number of stained particles, which are spanning larger size range when compared to control w-. (B) Quantification of absolute GFP:FYVE signal of perinuclear and cytoplasmic areas of Fig 2A. vap cells has significant elevations of the dispersed cytoplasmic PI(3)P pool (p<0.001) and perinuclear PI(3)P pool (p<0.05), both in the fed and starved conditions as compared respective controls. When subjected to starvation, control and mutant cells show a 3–4 time elevation of the GFP:FYVE signal (p<0,0001), indicating that starvation per se resulted in expanding PI(3)P-membrane influx. Error bars are mean differences; significances are from Student’s t-tests. (C) Determination of total PI(3)P phosphoinositide by Competitive ELISA using whole lipid extracts of fed and 3h-starved mid-3rd larvae (Materials and Methods). Absolute estimates in the mutant, vap1 animals are greater than those found in three reference strains used as controls: carnation (car1), Oregon-R (Or) and white (w-) (n = 3 except for car, n = 2). Each assay were the equivalent to 2.5 larvae. Error bars are standard deviations; significances are from Student’s t-tests. Genotypes. (C) Control: car1/Y. +/Y (Oregon-R). w1118/Y. Assay: vap1/Y. (D,D’) The size distribution of starvation-induced p62 bodies and Rab7:GFP aggregates of Fig 4B were analyzed in boxplots. p62 bodies and Rab7:GFP aggregates of mutant, vap1 cells are of a larger size range compared to control, w- cells. p62 bodies: (w- n = 357, Mdn = 0,27 μm2; vap1 n = 741, Mdn = 0.43 μm2). Rab7:GFP (w- n = 122, Mdn = 0.57 μm2; vap1 n = 367, Mdn = 0.67 μm2). Medians are drawn as thick lines; significances are from Mann Whitney test. (TIF) [file pone.0209759.s001.tif]

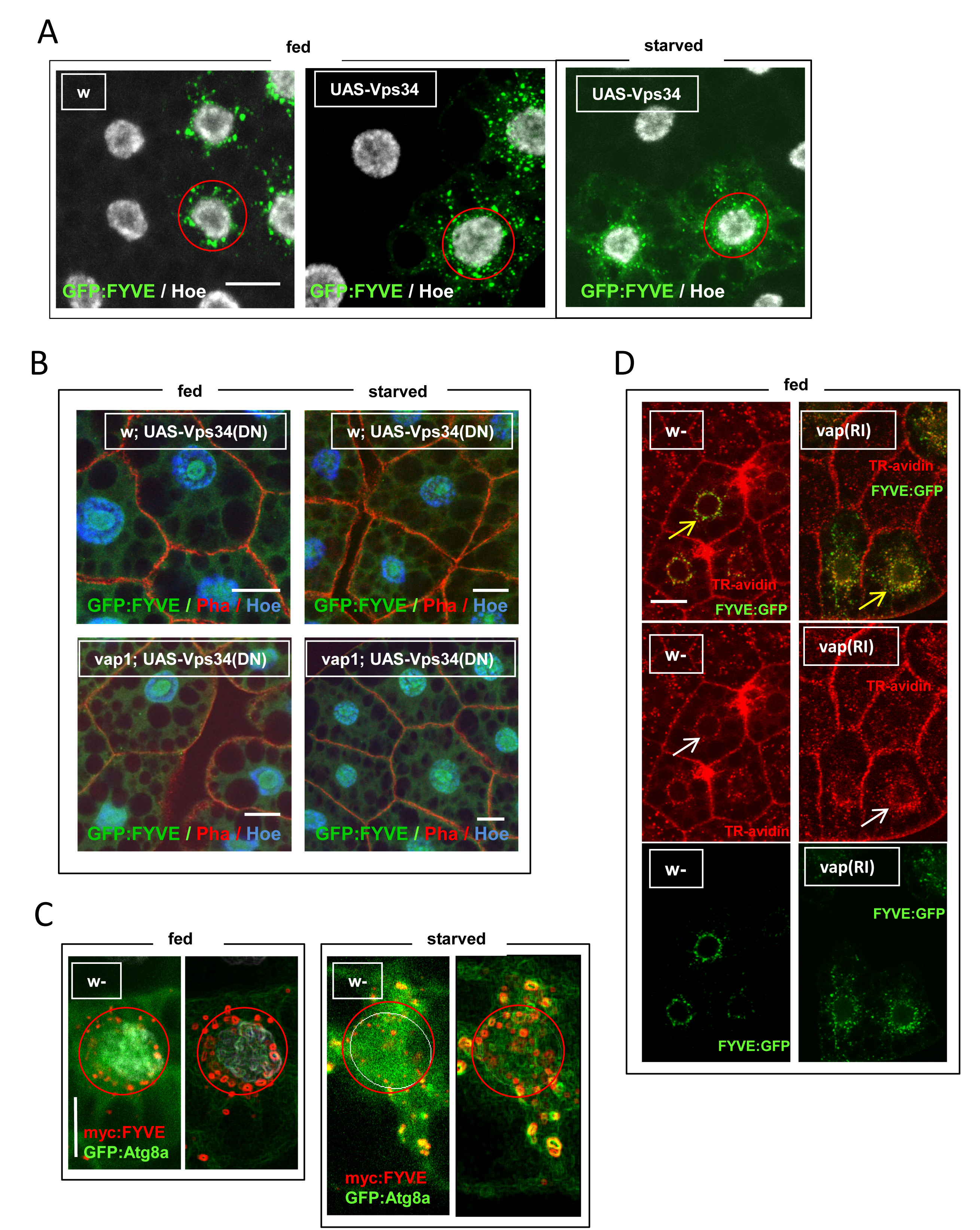

Supplement: S2 Fig — (A) Clonal overexpression of wild-type UAS-dVps34 transgene using the Act>CD2>Gal4 flipout cassette method, causes wider dispersion of PI(3)P in fed and 3h-starved cells compared to control, w-, as revealed by co-expressed GFP:FYVE biosensor probe. These effects are phenocopies of the loss of vap in fed and starved fat cells respectively (see Fig 2A). Scale bar = 20μm. (B) Inhibition of Vps34 using cg-Gal4-driven expression of dominant negative Vps34 (UAS-dVps34DN) in fat cells abolishes co-expressed GFP:FYVE staining in control, w- and vap1 contexts, both in fed and in1h30’-starved cells. Scale bars = 20μm. (C) Quantification of perinuclear versus cytoplasmic areas of stained FYVE probe was performed following a setup described in Juhàsz et al. [34] and illustrated here. Shown are GFP:Atg8a and myc(only)-tagged FYVE expressed in fed or starved fat cells, after immunostaining detection of myc (in red). In fed cells, the manually delimited red ring (2–4 μm around nuclei) comprised the perinuclear early endosomes. In starved cells, the delimiting red ring isolated inner endosomes from outer green and red labeled autophagosomes forming in the cytosol. When autophagosomes are not labeled, this method practically distinguished the two FYVE probe-labeled populations with about 90% accuracy. Images on the right were manipulated to enhance the stained structures. Scale bar = 10μm. (D) Clones of control, w- or vap RNAi-depleted cells, vap(RI), were generated in the presence of the GFP:FYVE probe expression, using the Act>CD2>Gal4 flipout cassette method and tissue subjected to ex-vivo TR-avidin incorporation (Materials and Methods). vap(RI) cells (marked by the GFP:FYVE) has increased labeled TR-avidin accessible compartment or perinuclear early endosomes (white arrows). Arrows in yellow point to the near complete overlap of the labeled tracer (red) and GFP:FYVE-labeled early endosomes (green) in control and mutant cells. Scale bar = 20 μm. Genotypes. (A) Control: w1118/ hsF [file pone.0209759.s002.tif]

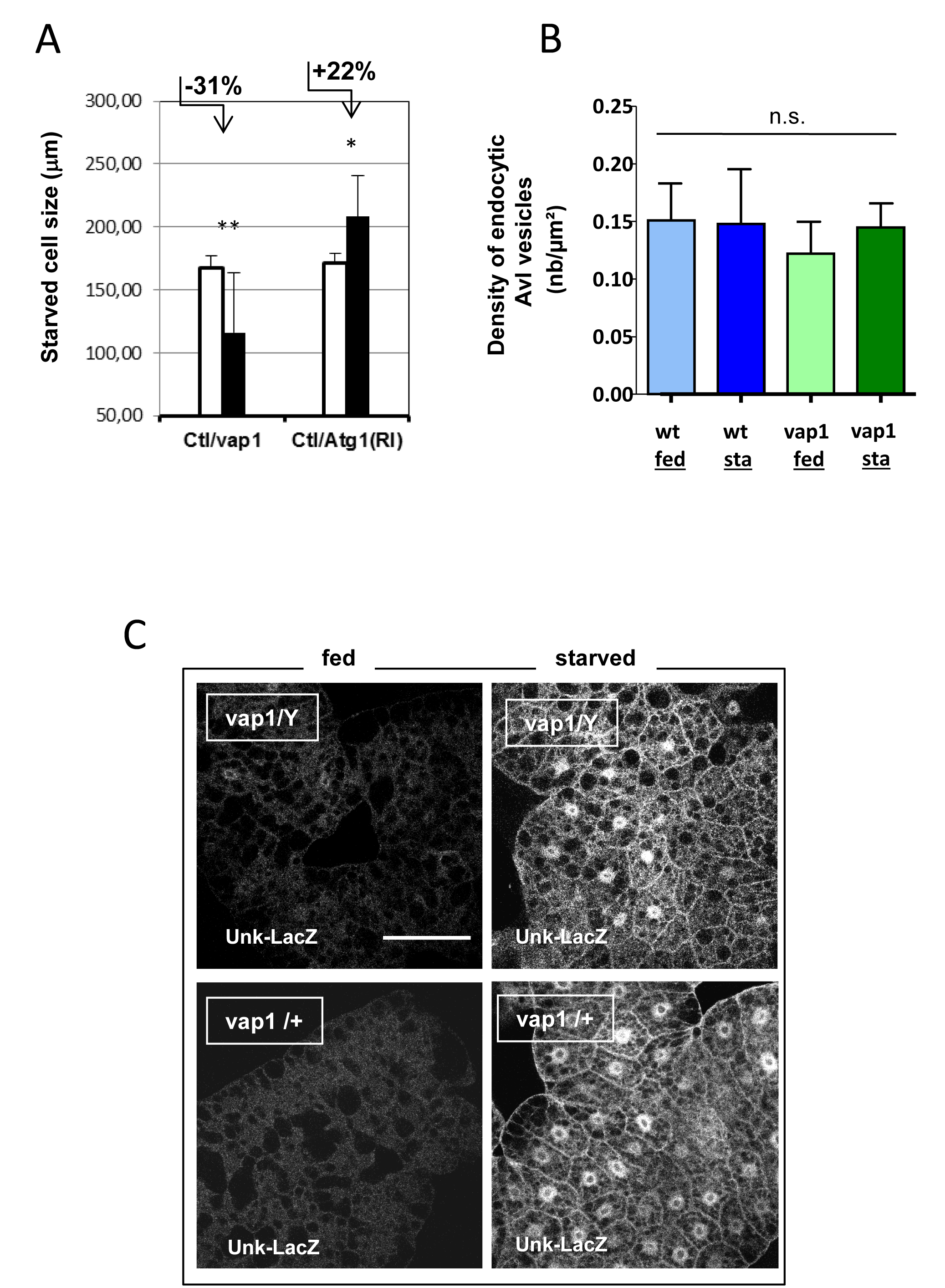

Supplement: S3 Fig — (A) Compared to clonal growth in fed conditions (Fig 3A and 3E), the relative size reduction of clonal vap1 fat cells versus control is not markedly different when animals grew under chronic starvation for ca. 88h (i.e. aa-poor food, Materials and Methods). Clones of Atg1(RI) mutant fat cells were analyzed in animal grown under the same chronic starvation for ca.88h. Atg1(RI) cells in this case, shows competitive growth advantage compared to control neighboring cells, as expected from autophagy-defective cells under starvation [14]. This data verified our chronic starvation conditions and the Atg1(RI) lines used in Fig 3. (Ctl n = 16, vap1 n = 8; Ctl n = 14, Atg1(RI) n = 13). Genotypes were as in Fig 3. Error bars are mean differences; significances are from Student’s t-tests. (B) Average populations of endocytic-compartment vesicles were analyzed after labeling for syntaxin 7/12 / D.melanogaster Avalanche (Avl). Densities of endocytic Avl vesicles (number of vesicle per μm2 of cell area) were quantified form plan surface views of control w- and vap1 fat cells, in fed and starved conditions as in Fig 8B–8C’. Avl-positive vesicle densities remains relatively even after starvation in control, w- or vap mutant conditions (wt (w-) fed n = 2; wt (w-) sta n = 2; vap1 fed n = 4; vap1 sta n = 3). Error bars are standard errors; significances are from ANOVA. (C) The activation of the unk-LacZ reporter construct was used to search for any devaluation of TOR-signaling in fat bodies of fed mutant vap animals. Images are immunostaining detection of LacZ expression. No staining of the reporter is observed in fed vap1/Y males larvae. On the other hand, reporter activation is readily obtained in tissue of 4h-starved mutant animals, attesting for normal inhibition of TOR-signaling and thus activation of the stress response factor REPTOR, which in turn mediates unk transcription [56]. Both negative and positive controls were obtained using fat bodies of hetererozygous, vap1/+ females [file pone.0209759.s003.tif]

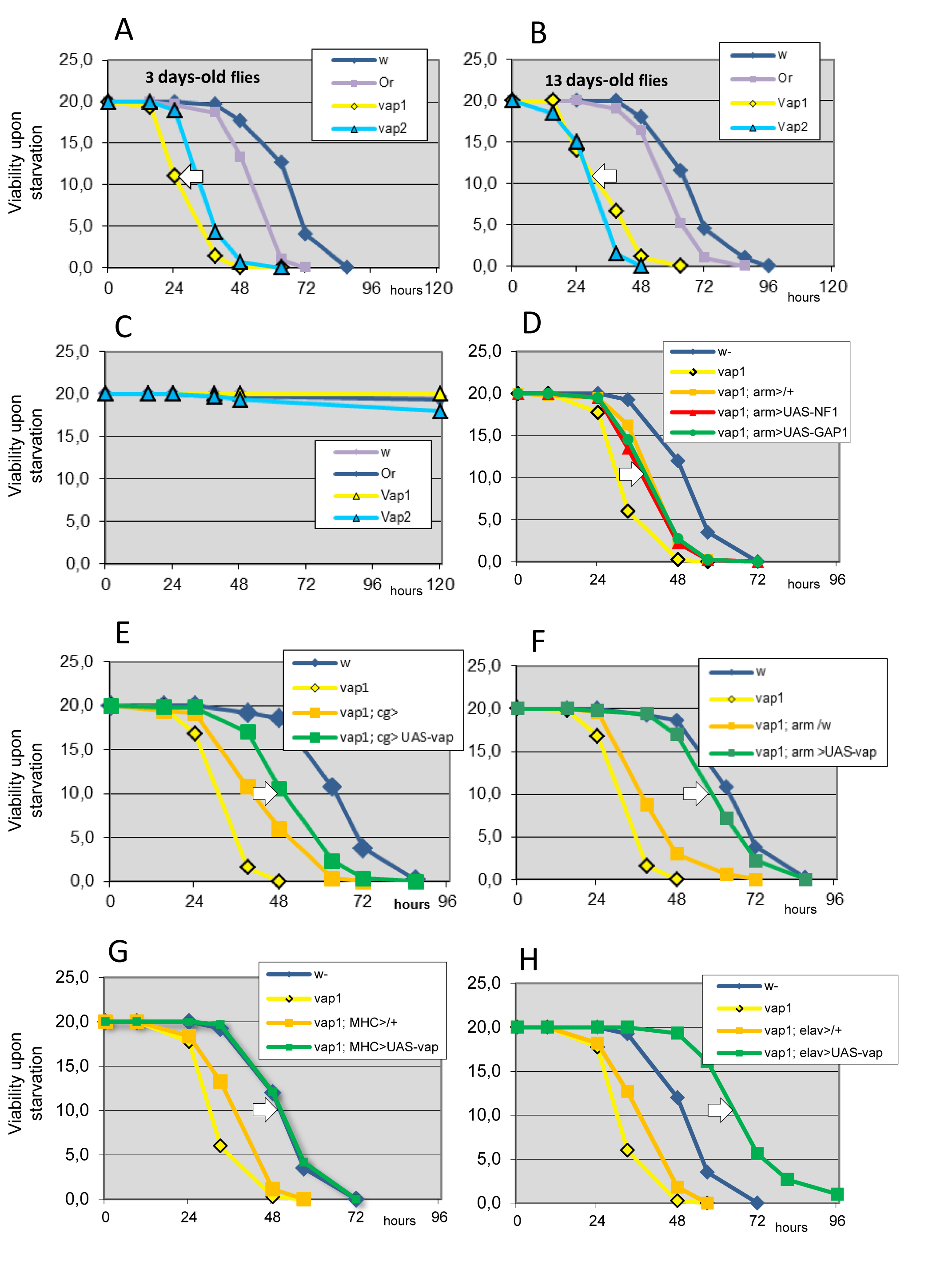

Supplement: S4 Fig — (A) Aged-matched, 3-days old mutant, vap1 and vap2 males exhibited robust hypersensitivity to acute starvation (white arrow), as 50% of them are not surviving for longer than 36h (see Materials and Methods for assay). Control, w- and Oregon-R, Or strains resist for a longer period. Female genotypes showed the same effects. (B) 13 days-old vap mutant flies shows an hypersensitivity-to-starvation phenotype similar to 3-days old flies despite the fact that mutant state has induced brain neurodegeneration for already 6 days in these flies [46]. (C) Flies fed with 15% sucrose only, are relieved from sensitivity to starvation whether mutants or controls. (D) Two D.melanogaster RasGAP family members distinct of Vap are ineffective at rescuing starvation sensitivity of vap1 mutants (D, white arrows) when expressed using the ubiquitous arm-Gal4 driver and corresponding transgenic wild-type constructs, UAS-NF1 and UAS-GAP1 [44]. (E-H) Partial rescue of starvation sensitivity is obtained after the restitution of vap activity using an UAS-vap(wt) transgene, and the fat body/hemocyte driver cg-Gal4 (E, white arrow). The same relative rescue is observed using the fat body/midgut driver ppl-Gal4 (not shown). Wider expression of vap using the arm-Gal4 driver, effect an almost complete rescue (F, white arrow). The same result is obtained using the ubiquitous driver Act-Gal4 (not shown). A complete rescue is associated with pan-muscular expression of vap using the Mhc-Gal4 driver (G, white arrow) whereas pan-neuronal expression of vap as driven by elav-Gal4, results in a rescue exceeding the limit of the w- controls suggesting enhanced resistance to starvation (H, white arrow). Genotypes. (A, B, C) Control: +/Y (Oregon-R). w1118/Y. Assay: vap1/Y. vap2/Y. (D-H) Included w1118/Y. vap1/Y. as control reference (D) Control: vap1/Y; arm-GAL4/+. Assay: vap1/Y; arm-GAL4/ UAS-NF1/+. vap1 /Y; arm-GAL4/ UAS-GAP1/+. (E) Control: vap1/Y; ppl-GAL4/+. Assay: vap1/Y; ppl-GAL4/ UAS- Vap:myc16.4/+. (F [file pone.0209759.s004.tif]

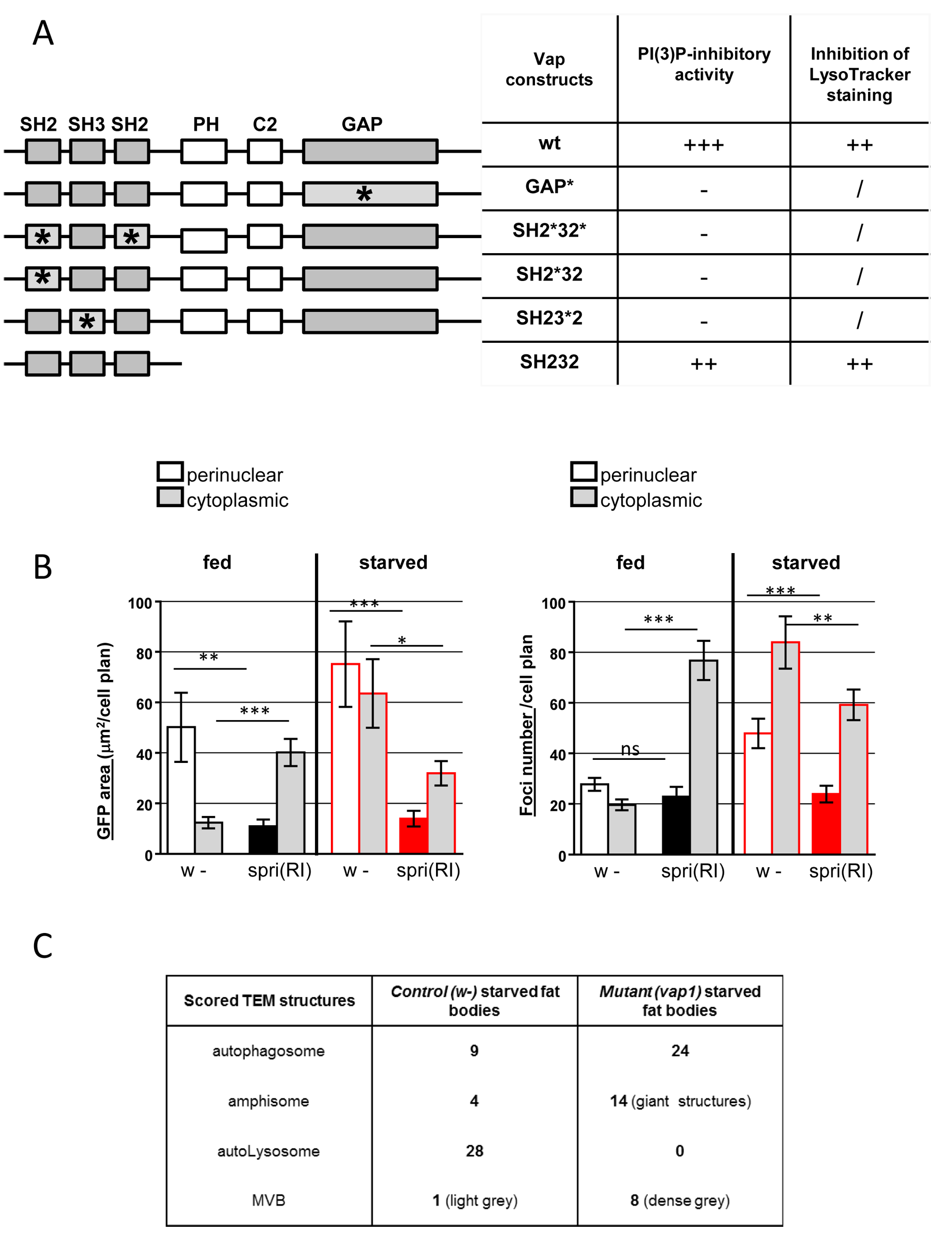

Supplement: S5 Fig — (A) A structure-function analysis of Vap domain mutants was performed based on the capacity of over-expressed Vap to antagonize fat body-cell PI(3)P as shown in Fig 2B. For each expressed constructed, a visual estimates of PI(3)P-inhibition was determined using 3 images of 3–4 cells wide clones. Maximal inhibitory activity of wild-type Vap (wt) is denoted as (+++), while GAP catalytic mutant GAP*, and the SH2*32*, SH2*32 and SH23*2 mutants are inactive at eliminating PI(3)P vesicles, and thus denoted as (-). The N-terminal fragment SH232, comprising the two SH2 domains is almost as active as wild type, which is denoted as (++). Activity of Vap (wt) and of the SH232 construct also inhibited LysoTracker staining (Fig 2C). Together, these analysis shows that SH2-domains are necessary and sufficient to suppress PI(3)P formation in fat body cells. Note that integrity of the GAP catalytic domain is essential for PI(3)P-inhibitory activity only when mutated as part of the full length protein. This is in accordance with the “Ras-effector model” of signaling proposed for human p120RasGAP activation [1*, 2*]. (B) Quantification of GFP:FYVE signals of perinuclear and cytoplasmic PI(3)P pools from Fig 6E (performed as in Fig 2). Mean GFP area signals (left chart) or mean foci number (right chart) of selected cells were calculate for w- control clones (fed n = 8 plans of 4 cells; sta n = 10 plans of 5 cells) and spri(RI) clones (fed n = 12 plans of 6 cells; sta n = 10 plans of 5 cells). The difference between the two representations is accounted by the small size of the spri- foci. Error bars are standard errors; Significances are from Student’s t-tests. (C) Characteristic vesicular autophagy-structures were compared in equivalent number of TEM sections of control, w- and vap1 starved fat tissues (Materials and Methods and Fig 4D). Note the accumulation of autophagosomes and amphisomes structures in the vap1 mutant, consistent with a block of the autophagy flux at late maturatio [file pone.0209759.s005.tif]

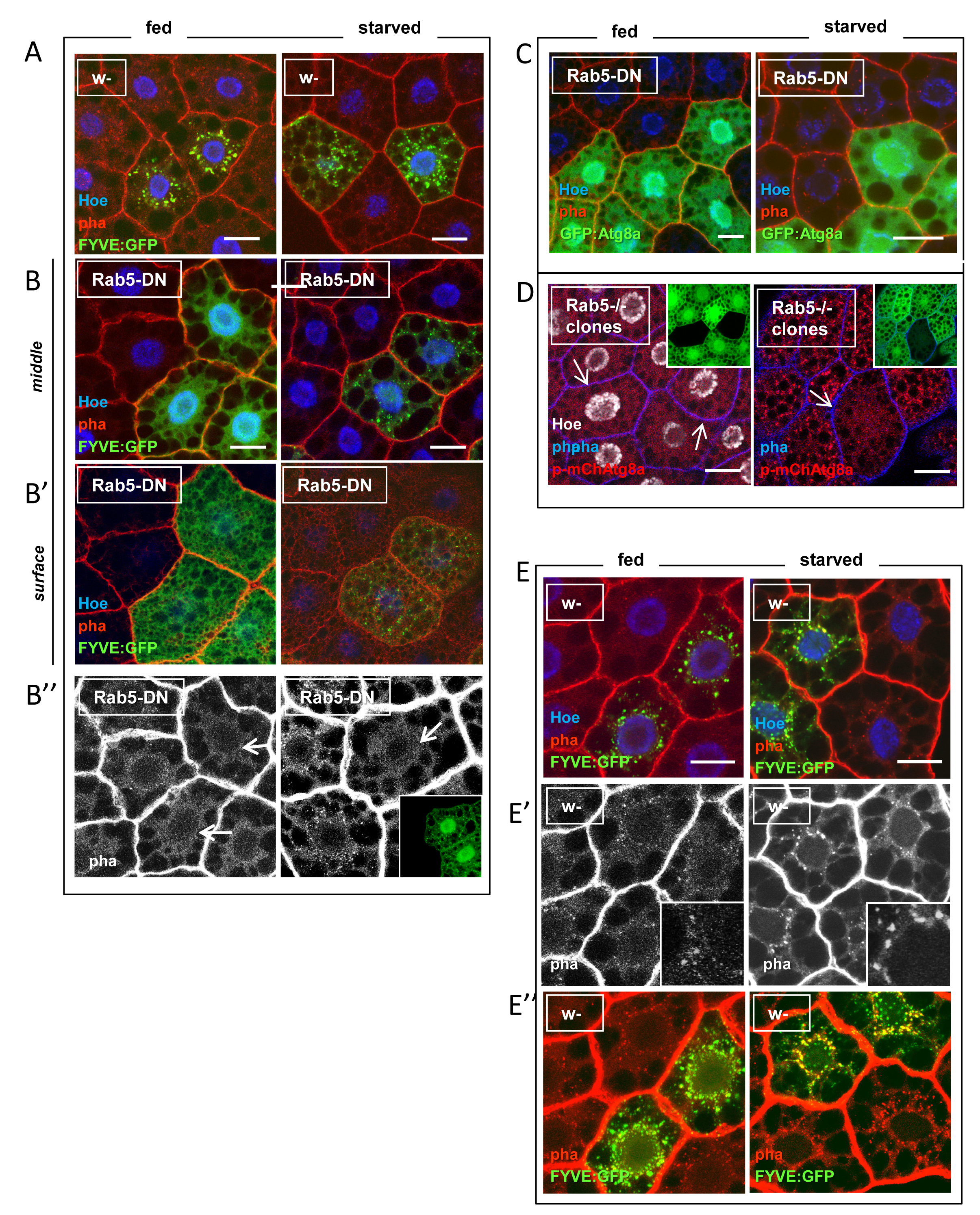

Supplement: S6 Fig — (A-B”) Control, w- and dominant-negative Rab5 expressing cell clones were generated using the Act>CD2>Gal4 flipout cassette method together with the GFP:FYVE marker. Fed or 3h-starved fat cells of early mid-3rd larvae were analyzed in fixed tissues focusing either in middle cell or below the cell surface close to cortical actin. Control, w- clones shows normal PI(3) P distribution in fed or starved fat cells (A). In fed cells, Rab5-DN prevents the formation of PI(3)P at pericentral and cell surface locations. Instead, fluorescent GFP:FYVE probe remains throughout the cytosol. In starved cells, residual PI(3)P vesicles are apparent in the cytosol and in the cell surface (B-B’). Rab5-DN clones are missing the pericentral actin network in fed and starved cells (arrows) as analyzed by phalloïdin staining (B”). (C) Rab5-DN clones are associated with expression of GFP:Atg8a marker (image from Fig 9D). No GFP-labeled autophagosomes are detected in the starved Rab5-DN expressing cells and fluorescent probe remains over the cytosol (Fig 4A and Fig 6A for GFP-labeled autophagosomes). (D) Rab52-null fat-cell clones were generated by mitotic recombination as in Fig 9A, in fed and 3h-starved animals and analyzed for the presence of mCherry-labeled autophagosomes using p-mChAtg8a made of the natural Atg8a promoter (Materials and Methods) (insert: identified GFP-, Rab5-/- clones). Not any red-labeled autophagosomes are present in fed cells and rare mCherry-labeled structures are associated with the Rab5-/- clone in starved cells (arrows) compared to neighboring wild-type cells. Note the large-sized Rab5 mutant cells in A through D. (E-E”) Control, w- cell clones is associated with GFP:FYVE marker expression and analyzed for phalloïdin staining, revealing thin pericentral punctuate structures in all fed cells and larger ones in all 3h-starved cells (E’ inset: higher magnification images). Image stacks helps to visualized the actin structures in fed cells (E”). Scale bars in all pan [file pone.0209759.s006.tif]

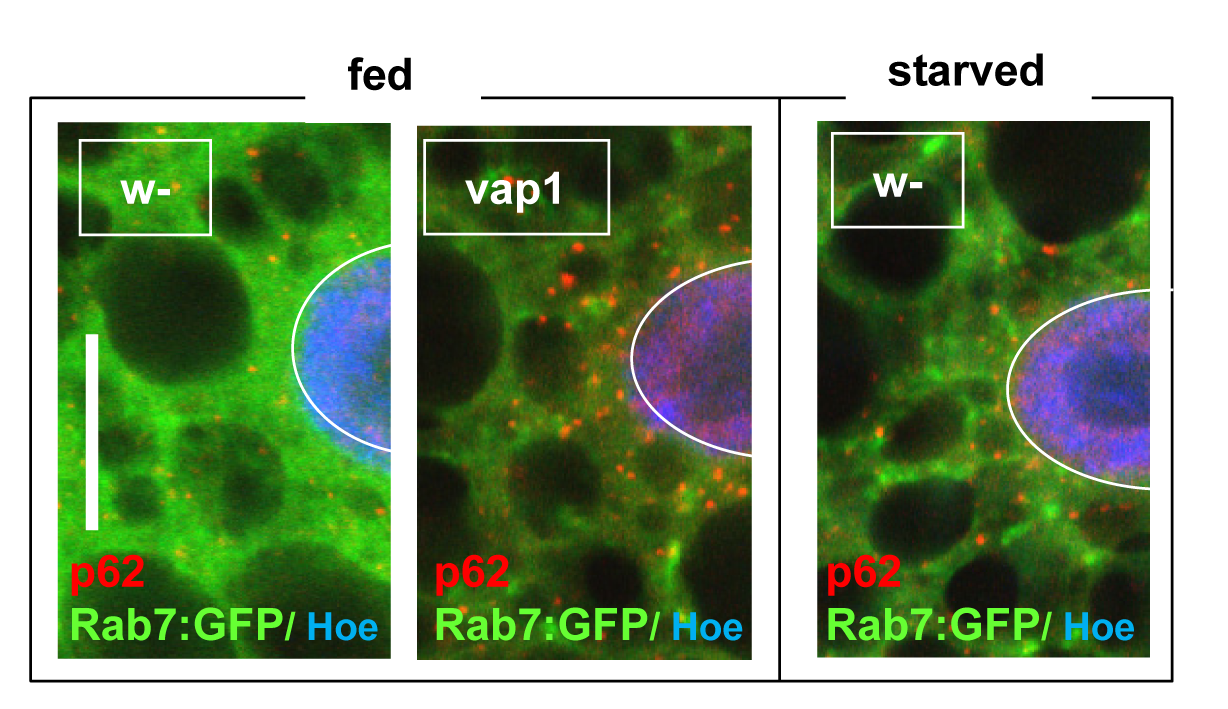

Supplement: S7 Fig — The state of endogenous p62/SQSTM1 autophagy-flux marker and late endosome Rab7:GFP marker were recorded in well fed cells of control, w- and mutant, vap1 fat bodies as in Fig 4B. These were compared to starved control, w- cells (image from Fig 4B). Fed mutant and starved control cells shows elevated number of p62 bodies and non-overlapping aggregates of Rab7:GFP, suggesting that autophagy is at least partially stimulated in the fed vap mutant conditions. Scale bar = 10 μm. (TIF) [file pone.0209759.s007.tif]

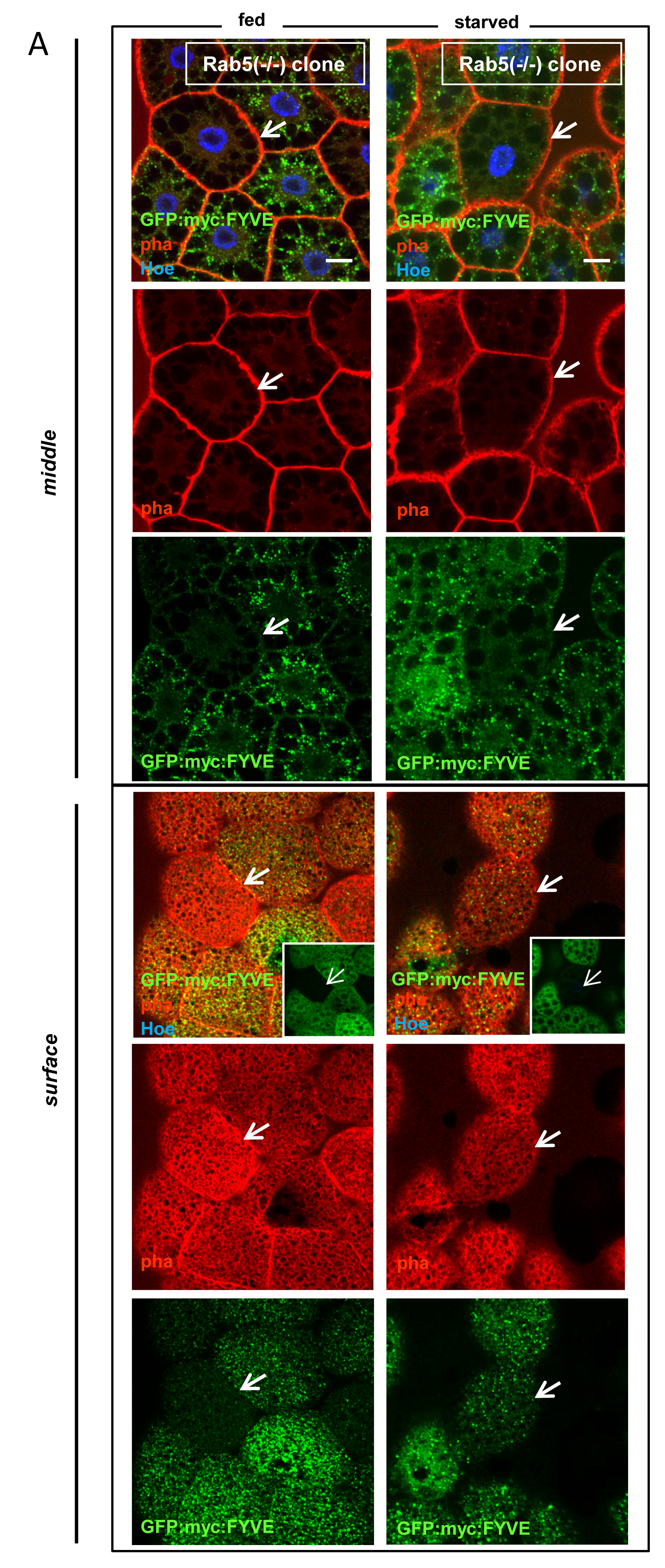

Supplement: S8 Fig — Scale bars = 20 μm. (TIF) [file pone.0209759.s008.tif]

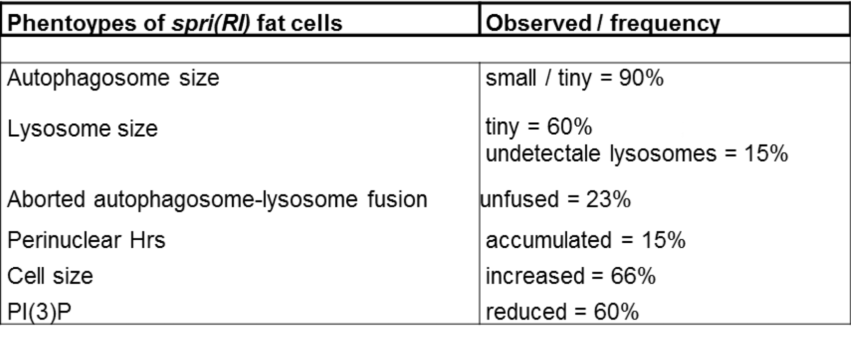

Supplement: S1 Table — Note that analyzed autophagy defects of spri(RI) fat cells were always showing incomplete penetrance (n = 30–40 analyzed cells). This feature is likely in relation to the variegating nature of the spri gene [52] or the RNAi construct. (TIF) [file pone.0209759.s009.tif]
